# Impact of China’s Low Centralized Medicine Procurement Prices on the Cost-Effectiveness of Statins for the Primary Prevention of Atherosclerotic Cardiovascular Disease

**DOI:** 10.5334/gh.830

**Published:** 2020-06-25

**Authors:** Miao Wang, Jing Liu, Brandon K. Bellows, Yue Qi, Jiayi Sun, Jun Liu, Andrew E. Moran, Dong Zhao

**Affiliations:** 1 Department of Epidemiology, Beijing Anzhen Hospital, Capital Medical University, Beijing Institute of Heart, Lung and Blood Vessel Diseases, Beijing, CN; 2 The Key Laboratory of Remodeling-Related Cardiovascular Diseases, Ministry of Education, Beijing, CN; 3 The Beijing Municipal Key Laboratory of Clinical Epidemiology, Beijing, CN; 4 Division of General Medicine, Department of Medicine, Columbia University, New York, US

**Keywords:** cardiovascular disease, cost-effectiveness, low-density lipoprotein cholesterol, microsimulation, primary prevention, statins

## Abstract

**Background:**

Statin medications reduce the risk of atherosclerotic cardiovascular disease (ASCVD). China’s new central government medicine procurement policy lowered statin prices by five-fold or more, which may impact the cost-effectiveness of statin therapy.

**Objective:**

To explore the impact of China’s 2019 centralized medicine procurement policy on the cost-effectiveness of statins treatment for primary ASCVD prevention.

**Methods:**

A microsimulation decision tree analytic model was built using individual participant data from ASCVD-free adults aged 35–64 years (n = 21,265) in the China Multi-provincial Cohort Study. ASCVD incidence, costs (2019 Int$), and quality-adjusted life years (QALYs) over a 10-year period from health-care sector and societal perspectives were estimated. Effect and cost-effectiveness of low-dose statins (equivalent potency regimens of simvastatin 20 mg/day, atorvastatin 10 mg/day, or rosuvastatin 5 mg/day) and moderate-dose (double low dose) statins therapy were simulated. The incremental cost-effectiveness ratio (ICER) of statin treatment was compared with no treatment by category of 10-year ASCVD risk. New lower prices of statins were from the centralized procurement policy bid-winning announcement file. One-way and probabilistic sensitivity analyses quantified model uncertainty.

**Results:**

Low-dose statins interventions reduced 10-year ASCVD incidence by 4.1%, 9.7%, and 15.5% among people with low, moderate, and high risk comparing to no treatment. Lowering statin prices to the 2019 central government procurement policy level could lower the ICER of low-dose statins treatment for high-risk people from Int$ 141,000 to Int$ 51,300 per QALY gained from health-care sector perspective. Moderate-dose statin treatment lowered the ICER compared with the low-dose statins treatment in each ASCVD risk category (Int$ 43,100 vs. Int$ 51,300 per QALY gained from the health-care sector perspective for high risk people). Cost-effectiveness improved progressively with increased baseline ASCVD risk.

**Conclusion:**

Implementing low central government prices will substantially improve the cost-effectiveness of statins for primary ASCVD prevention in 35–64-year-old Chinese adults.

Atherosclerotic cardiovascular disease (ASCVD) remains the leading cause of death in China, where over one-fifth of the world’s ASCVD events occur each year [1,2]. China’s evidence-based lipid management guideline recommends using lipid-lowering therapy with statins to control lipids and prevent ASCVD for people with high risk of ASCVD and recommends controlling low-density lipoprotein cholesterol (LDL-C) at lower than 3.4 mmol/L for people with moderate or low risk [3]. However, statins remain underused for Chinese adults meeting treatment criteria [4,5].

In Western countries, statin therapy is cost-effective or cost-saving, especially in people with a high baseline ASCVD risk [6,7,8,9,10,11,12]. Statin medication costs are a key determinant of cost-effectiveness [7]. In high-income Western countries, statin medicine costs are relatively low, while acute and chronic treatment costs for ASCVD are high, which explains why statins are so cost-effective in those countries [7,9,10,11]. In China, however, until recently statins costs were relatively high and ASCVD treatment costs relatively low, which together made for a less favorable cost-effectiveness ratio.

In 2019, China pursued a new centralized medicine procurement policy, which aims to lower medicine prices through competitive bidding, bulk purchasing, and reduced transaction costs. The pilot program of the new policy implemented in the ‘4 + 7 cities’ (Beijing, Tianjin, Shanghai, Chongqing, Shenyang, Dalian, Xiamen, Guangzhou, Shenzhen, Chengdu, and Xi’an) successfully lowered medicine prices at the point of purchase and reduced patient out-of-pocket drug costs. For example, the average annual medicine fee of daily low-dose statins was reduced from approximately 284 Int$ to 48 Int$ (Table 1) [13,14]. This computer simulation study sought to estimate the effectiveness and cost-effectiveness of statins for primary ASCVD prevention in Chinese adults aged 35–64 years at different 10-year ASCVD risk thresholds, and to explore the potential impact of new lower statin prices resulting from China’s new centralized medicine procurement policy.

**Table 1:** Selected parameters used in this simulation study.

Category	Mean	Variance (SE)	Lower limit of 95 CI%	Upper limited of 95 CI%	Distribution	Source

**Effect of statin treatment on LDL-C change (%)**						
Low-dose statins*	–30.0	—†	—†	—†	—	American Heart Association/American College of Cardiology Cholesterol Management Guideline [17]
Moderate-dose statins‡	–49.0	—†	—†	—†	—	
**Relative change in ASCVD risk per mmol/L LDL-C reduction (%)**						
Acute coronary events§	–29.0	—	–35.0	–23.0	Log-normal	Meta-analysis of Individual Data from the Cholesterol Treatment Trialists’ (CTT) Collaborators [18]
Ischemic stroke§	–21.0	—	–26.0	–15.0	Log-normal	
**Direct costs**						
Yearly costs for statins before the new centralized medicine procurement policy was implemented						
Low-dose statin (Int$/year)	284.2	117.8	68.9	530.5	Gamma	Integrated Management Platform of Beijing Medicine Sunshine Purchase [13]
Moderate-dose statin (Int$/year)	568.4	235.5	137.8	1061.0	Gamma	
Yearly costs for statins under the new centralized medicine procurement policy						
Low-dose statin (Int$/year)	48.2	7.0	34.6	61.9	Gamma	National centralized procurement policy bid-winning announcement file
Moderate-dose statin (Int$/year)	96.5	13.9	69.2	123.8	Gamma	
Lowest cost for low-dose statin (Int$/year)	40.2	—†	—†	—†	—	
Statin treatment related costs						
Examination for risk assessment of ASCVD (Int$/time) ||	19.3	1.5	16.4	22.3	Gamma	—
Safety screening in the first year (Int$/year) ||	139.2	10.8	118.2	160.3	Gamma	—
Safety screening after first year (Int$/year) ||	121.8	9.4	103.4	140.3	Gamma	—
Yearly costs for diabetes treatment (Int$/year)	518.5	40.1	439.9	597.0	Gamma	China’s study on medical expenditure of diabetes mellitus [19]
Costs for rhabdomyolysis (Int$/visit)	2512.2	400.4	1727.3	3297.1	Gamma	China’s Health and Family Planning Statistical Yearbook [20]
Annual health-care and ASCVD treatment costs						
Annual health-care costs (Int$/year)	947.1	73.2	803.6	1090.6	Gamma	China’s Health and Family Planning Statistical Yearbook [20]
Hospitalization costs for acute coronary events (Int$/visit)	8384.0	1336.4	5764.6	11003.4	Gamma	
Hospitalization costs for ischemic stroke (Int$/visit)	2923.3	642.6	1663.9	4182.7	Gamma	
First aid cost for acute coronary events (Int$/visit)	1413.8	225.4	972.1	1855.5	Gamma	
First aid cost for ischemic stroke (Int$/visit)	470.8	103.5	268.0	673.6	Gamma	
Yearly costs in chronic stage for acute coronary events (Int$/year)	1695.4	131.1	1438.5	1952.3	Gamma	China Health and Retirement Longitudinal Survey [21]
Yearly costs in chronic stage for ischemic stroke (Int$/year)	1417.9	109.6	1203.1	1632.7	Gamma	The Fifth National Health Services Survey in Henan Province [22]
Incidence of adverse events (%)						
Diabetes Low-dose statin Moderate-dose statin	0.6 1.2	—	0.42 0.92	0.85 1.52	Log-normal	HPS2-THRIVE study (Data of Chinese participants were used) [16]
Rhabdomyolysis Low-dose statin Moderate-dose statin	0.02 0.04	—	0 0	0.10 0.13	Log-normal	
Myopathy Low-dose statin Moderate-dose statin	0.22 0.44	—	0.13 0.31	0.41 0.70	Log-normal	
Other reason for stopping treatment non-adherence	12.4	—†	—†	—†	—†	

ASCVD, atherosclerotic cardiovascular disease; CI, confidence interval; SE, standard error; LDL-C, low-density lipoprotein cholesterol; QALY, quality-adjusted life-year; 1 Int$ = 3.539 Chinese Yuan (RMB) and all costs were inflated to 2019. *, Equivalent potency regimens of simvastatin 20 mg/day, atorvastatin 10 mg/day, or rosuvastatin 5 mg/day. †, Parameters in sensitivity analysis were unchanged. ‡, Equivalent potency regimens of simvastatin 40 mg/day, atorvastatin 20 mg/day, or rosuvastatin 10 mg/day. §, Relative risk change= (1-relative risk)*100% ||, The costs were estimated base on the examination items, test frequency, and costs. The costs of biochemical tests were from the Beijing Municipal Commission of Development and Reform.

## Methods

### Simulation model design

We constructed a microsimulation decision tree analytic model to estimate the incidence of ASCVD events, health-care costs, and quality-adjusted life years (QALYs) over a 10-year time horizon. We defined ASCVD as acute coronary events (i.e., myocardial infarction, cardiac arrest, and coronary death (International Classification of Diseases, 10th Revision [ICD-10] codes: I20–I25 and I46) and ischemic stroke (ICD-10 codes: I63 and I69.3). Costs were estimated from both a societal perspective (including direct and indirect costs) and from a health-care sector perspective (including only direct costs). Future costs and QALYs were discounted 3% annually by applying an average weight of 0.875 for cost and QALYs for the 10-year simulation in the decision tree model [11].

**Figure 1:**
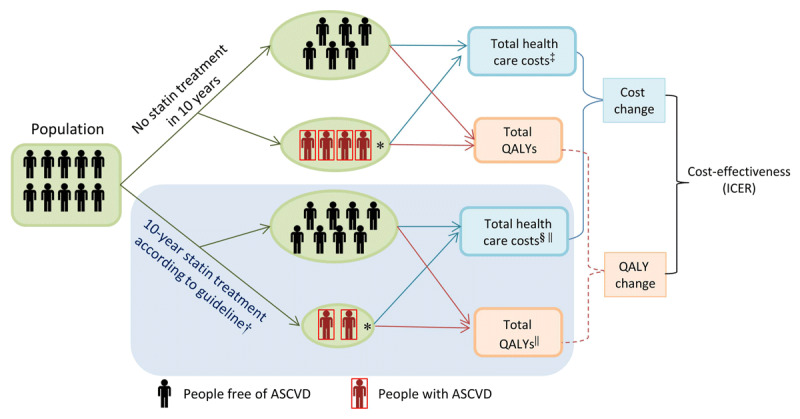
Conceptual diagram of the simulation model. ASCVD, atherosclerotic cardiovascular disease; QALYs, Quality adjusted life years, ICER, incremental cost effectiveness ratio. *, People suffered from ASCVD in 10 year among the cohort population. †, Less people developed ASCVD in the cohort after intervention compared to the scenario of no intervention. ‡, Costs before intervention included: per capita total expenditure on health, hospitalization and post-event outpatient ASCVD management costs, and indirect costs due to onset of ASCVD. §, Costs after intervention included: costs before intervention, statin treatment related cost and side effects treatment costs. ||, Under statin treatment, the statin treatment related adverse effects would decrease quality of life and incur treatment cost for people who receive statin treatment.

As shown in the model diagram (Figure 1), people without a history of ASCVD are at risk of developing ASCVD over the 10-year simulation, conditional on exposure to ASCVD risk factors. Details concerning the model structure can be found in the Supplementary Appendix and Online Figures 1 and 2. In the model, total non-ASCVD and ASCVD health-care costs and indirect costs were calculated for each patient by summing the product of the duration of time spent in each health state (i.e., no ASCVD, acute ASCVD, and chronic ASCVD) and the corresponding annual cost. Similarly, total QALYs were calculated by summing the product of the duration of each health state by the corresponding utility (i.e., quality of life weights ranging from zero, death, to one, perfect health). The incremental cost-effectiveness ratio (ICER) was calculated by dividing the change in mean costs by the change in mean QALYs.

Persons without ASCVD at baseline enter the model and are then eligible to experience one of several outcomes: no ASCVD event, fatal or non-fatal incident ASCVD, or non-ASCVD death (Online Figure 1). Every person aged 35–64 years old at baseline could survive for up to 10 years. Each person was first assigned a 10-year risk of ASCVD calculated using the Chinese 10-year ASCVD risk assessment equation, which was developed based on the China Multi-provincial Cohort Study (CMCS) data, conditional on their base year risk factors [15]. Then, predicted ASCVD events were categorized into either first acute coronary event or first ischemic stroke event based on the sex and age group-specific allocation proportions of these two types of disease observed in the CMCS. ASCVD fatalities could occur either within or after 30 days, and in or out of hospital. Both non-fatal and fatal hospitalized ASCVD patients were assumed to receive standard hospital treatments. Patients with non-fatal ASCVD additionally received outpatient secondary prevention treatment including statin treatment in the base case. Hospital and outpatient secondary prevention treatments were held constant across primary prevention scenarios we evaluated. We assumed people could have a maximum of two ASCVD events during 10-year simulation period. A proportion of individuals taking statins discontinued treatment due to statin intolerance or statin-related adverse events (Table 1) [16].

### Simulated treatment strategies

We explored the effect and cost-effectiveness of two doses of statins treatment implemented for eligible people by ASCVD risk level for China’s ASCVD primary prevention (Table 2). One was low-dose statins (i.e., simvastatin 20 mg/day, atorvastatin 10 mg/day, or rosuvastatin 5 mg/day) which lowered LDL-C by 30.0% (Table 1) [17,23]. The second was ‘moderate-dose’ statins, that is, double the dose of statins used in the first scenario, which decreased LDL-C by 49.0%. The two dose levels corresponded to Chinese and international statin guideline recommendations [3,17].

**Table 2:** Criterions and parameters of statin treatment recommendation for people with different risk.

	Total	Low risk	Moderate risk	High risk*

10-year ASCVD risk threshold	—	<5%	5.0–9.9%	≥10.0%
Guideline-recommended LDL-C intervention threshold (mmol/L)†	—	≥3.4	≥3.4	≥2.6
Proportion of people in each risk category in CMCS (%)	100.0	70.6	15.4	14.0
Proportion of people eligible for statin treatment in CMCS (%)‡	26.0	15.4	31.0	73.6

ASCVD, atherosclerotic cardiovascular disease; CMCS, Chinese Multi-provincial Cohort Study; LDL-C, low-density lipoprotein cholesterol. *, High-risk defined as: LDL-C ≥ 4.9 mmol/L or total cholesterol ≥ 7.2 mmol/L; diabetes and LDL-C 1.8–4.8 mmol/L or TC 3.1–7.2 mmol/L and age ≥ 40 years; or 10-year ASCVD risk ≥ 10%. †, Recommended by the 2016 Chinese Guidelines for the Management of Dyslipidemia in Adults. ‡, People met the guideline recommendation and without use of lipid-lowering agents in each risk category.

In the statin treatment arms, the risk of ASCVD events was decreased per mmol/L of LDL-C lowering using disease-specific β coefficients based on the meta-analysis from Cholesterol Treatment Trialists’ Collaboration (Table 1) [18]. In model simulations, statin treatment was also associated with a risk of medication-related adverse events, including diabetes, rhabdomyolysis, and myopathy, which incurred costs and utility decrements. Other potential statin adverse effects, including elevated liver enzymes, were assumed not to be clinically meaningful but could result in medicine discontinuation (Supplementary Appendix). We assumed those who discontinued statin treatment did so in the middle of the simulation time horizon, and no longer had a reduction in LDL-C or ASCVD risk.

### Simulation cohort

The simulation cohort was derived from the CMCS study, a nationwide, multi-center cohort study on the determinants of ASCVD incidence in China. Participants free of cardiovascular disease were enrolled at study baseline from 1992 to 1996 [24]. Informed consent was obtained from all participants. The baseline LDL-C levels and other risk factors of the 21,265 individual Chinese participants aged 35–64 years in the CMCS study were imported into the model to calculate the individual 10-year risk of ASCVD. The individual risk formed the incidence number of ASCVD and was also a criterion for selecting participants eligible for statin intervention under contemporary guidelines [3]. Details of the CMCS cohort can be found in the Supplementary Appendix (Online Table 1). This study was approved by the ethics committee of Beijing Anzhen Hospital, Capital Medical University (Approval Number: KS2016005).

### Risk stratification

High ASCVD risk was defined according to the recommendations of the Chinese Guidelines for the Management of Dyslipidemia in Adults as follows: 1) LDL-C ≥ 4.9 mmol/L or total cholesterol (TC) ≥ 7.2 mmol/L; 2) diabetes and LDL-C in the range of 1.8–4.8 mmol/L (or TC 3.1–7.2 mmol/L) and age ≥ 40 years; 3) 10-year ASCVD risk ≥ 10%. Otherwise, ASCVD risk was based only on a 10-year ASCVD risk score: 5%–9.9% was considered moderate risk and <5% was considered low risk [3].

### Costs

Health-care and ASCVD treatment costs were derived from the Fifth National Health Services Survey [19], China’s Health and Family Planning Statistical Yearbook [20], and other related studies (Table 1) [21,22]. For new ASCVD events, we included non-ASCVD health expenditures, ambulance costs, hospitalization cost, post-event outpatient ASCVD management costs, and indirect costs (Online Figure 2). Indirect costs, which estimated the financial losses in current and future years due to disability and death from ASCVD for the patients and their caregivers, included patient-time costs, unpaid caregiver-time costs, transportation costs, labor market earnings lost due to disease (Online Table 2) [25]. The average wage used in calculating indirect costs was based on wages of people working in non-private and private institute in 31 provinces (autonomous regions, municipalities) of China. More details concerning the indirect cost estimation method are provided in Online Table 3.

For statin treatment, we also included medication costs, routine lipid panel test costs, the cost of ASCVD risk assessment, medication-related adverse event monitoring, and adverse event treatment, if such events occurred. The yearly cost of statins was calculated by multiplying days in a year by the dose per day (mg/day), and the average cost per mg across the types of statins available in China (Int$/mg).

Prices for statins prior to implementation of the new centralized medicine procurement policy were obtained from the website of the Integrated Management Platform of Beijing Medicine Sunshine Purchase, which lists the prices of medicine in hospitals in Beijing prior to implementation of the new policy [13]. The lower statin prices that could be achieved after the implementation of the new policy in China were taken from the national centralized procurement policy bid-winning announcement file [14]. Annual statin treatment costs are listed in Table 1. All costs were inflated to 2019 using the rate of inflation in China published by Trading Economics and then converted into international dollars (Int$) according to the purchasing power parity exchange rate published by the Organization for Economic Co-Operation and Development (1 Int$ = 3.539 Chinese Yuan (RMB) in 2019; accessed 2/6/2020) [26,27]. Purchasing power parity exchange rates are based on each country’s prices for a package of standard goods and, unlike currency exchange rates, are not subject to central bank policy effects.

### Utilities

Every health state was assigned a utility which was used to estimate the benefits and harms of statin use on health in the model (Online Table 4 and Supplementary Appendix), including utilities for chronic and acute stage of ASCVD and adverse events [6,7,28,29].

### Model calibration and validation

The model was calibrated to achieve the best fit (minimal residual difference) between the simulation model’s predicted rate and the observed rate, including the person-year incidence of ASCVD and non-ASCVD mortality. Details were shown in the Supplementary Appendix. After calibration, the ratios of the simulated to observed rates were all close to one (Online Figure 3).

### Analysis

We compared the simulated incidence of ASCVD with no treatment to each statin treatment arm (i.e., the low- and moderate-dose statins) and calculated the number needed to treat to prevent one ASCVD event in 10 years (NNT_10_) and the QALYs gained. We used the projected incremental costs and QALYs to calculate the ICER, defined as the incremental cost to gain one additional QALY. In this study, the treatment effect in terms of preventing ASCVD and the budgetary impact of the statins price change stemming from the new policy were estimated on the basis that all eligible people aged 35–64 years old in China received statins for primary prevention. Details are shown in the Supplementary Appendix.

We used willingness-to-pay thresholds recommended by the World Health Organization’s CHOosing Interventions that are Cost Effective (WHO-CHOICE) initiative to determine the cost-effectiveness of statins in China. WHO-CHOICE defines an ICER that is less than the gross domestic product (GDP) per capita as highly cost-effective; an ICER that is 1–3 times GDP per capita as moderately cost-effective; and an ICER more than three times GDP per capita as not cost-effective. The GDP per capita of China in 2018 was Int$ 18,266 (64,644 Chinese Yuan) [30].

One-way sensitivity analyses explored the impact of statin prices, utilities, and adverse event incidence rates on the cost-effectiveness estimates. Monte Carlo probabilistic sensitivity analyses randomly sampled the uncertainty distributions of three key sets of inputs simultaneously: the relative risk of ASCVD events per mmol/L LDL-C change, all direct and indirect cost inputs, and the incidence rate of three types of adverse events. In the analysis, the distributions of these inputs were randomly sampled 1,000 times, resulting in 1,000 pairs of total costs and QALYs that were used to calculate the proportion of ICERs falling under our predetermined willingness-to-pay threshold.

The China statin decision ASCVD microsimulation model was developed in SAS software (version 9.4, SAS Institute, Cary, NC, USA). The SAS CALL STREAMINIT function was used to generate log normal distributions of treatment effects and adverse event parameters for probabilistic sensitivity analysis. The SAS MCMC function was used to create gamma distributions for costs. This study was designed and reported according to the CHEERS statement [31].

## Results

### ASCVD incidence

The 10-year cumulative incidence rates of ASCVD among low, moderate, and high risk people were 1.8%, 6.9%, and 10.7%, respectively, in CMCS cohort population. Compared with no statin treatment, the low-dose statin treatment resulted in relative reductions of the ASCVD incidence by 4.1% (95% CI: 3.2%–5.2%), 6.4% (95% CI: 5.1%–8.3%), and 15.5% (95% CI: 12.2%–20.2%) among people with baseline low, moderate, and high 10-year ASCVD risk, respectively (Table 3 and Online Table 5). These reductions could be translated into reductions in 10-year ASCVD incidence among the low, moderate, and high risk populations in China of 323,000 (95% CI: 253,000–413,000), 399,000 (95% CI: 317,000–521,000), and 1,350,000 (95% CI: 1,083,000–1,748,000), respectively (Table 4). With the moderate-dose statin treatment, a larger relative reduction in ASCVD incidence would be achieved among all ASCVD risk groups.

**Table 3:** Cost-effectiveness of statin treatment vs. no statin treatment over 10 years stratified by ASCVD risk from health-care sector perspective and using statin prices from the new centralized medicine procurement policy.

ASCVD Risk	Low-dose statin strategy*			Moderate-dose statin strategy**		

	Low risk	Moderate risk	High risk	Low risk	Moderate risk	High risk

**Relative reduction of ASCVD incidence (%)**	4.1 (3.2,5.2)	6.4 (5.1,8.3)	15.5 (12.2,20.2)	6.2 (4.9,7.8)	9.7 (7.8,12.3)	23.4 (19.1, 29.8)
**NNT10**	193.0 (150.6, 246.0)	67.3 (51.7, 84.4)	42.8 (33.0, 53.4)	126.8 (101.0, 159.1)	44.3 (34.7, 54.6)	28.4 (22.3, 34.8)
**Average 10-year treatment cost for each eligible person (Int $)‡**	15,00 (1,200, 1,800)	1,200 (1,000, 1,600)	1,100 (900, 1,400)	1,800 (1,400, 2,300)	1,600 (1,200, 2,000)	1,400 (1,100, 1,800)
**ICER (Int$/QALY)**	380,700	92,300	51,300	347,500	78,000	43,100
**Probability of highly cost-effectiveness (%)**	0	0	0	0	0	0
**Probability of cost-effectiveness (%)**	0	0.2	64.9	0	2.6	90.8

ASCVD, atherosclerotic cardiovascular disease; NNT10, number needed to treat for 10-year intervention; QALY, quality adjusted life year. *, Equivalent potency regimens of simvastatin 20 mg/day, atorvastatin 10 mg/day, or rosuvastatin 5 mg/day. **, Equivalent potency regimens of simvastatin 40 mg/day, atorvastatin 20 mg/day, or rosuvastatin 10 mg/day. ‡, Estimated base on direct cost such as change of ASCVD treatment cost due to decrease of ASCVD incidence, statin medicine cost expenditure, statin treatment related cost, routine health-care cost, and adverse events treatment cost. Costs are in 2019 International dollars (Int$). Highly cost-effective: cost per QALY gained was in the range between Int$ 0 and Int$ 18,266. Cost-effective: cost per QALY gained was in the range between Int$ 18,267 and Int$ 54,798.

**Table 4:** Estimated 10-year treatment effect and direct costs change among 35–64 years old ASCVD-free people if the new centralized medicine procurement policy implemented in the whole nation.

ASCVD risk Category	Low-dose statin strategy			Moderate-dose statin strategy		

	Low risk	Moderate risk	High risk	Low risk	Moderate risk	High risk

**Prevented incidence number of ASCVD (N)**	323,000 (253,000, 413,000)	399,000 (317,000, 521,000)	1,350,000 (1,083,000, 1,748,000)	492,000 (391,000, 617,000)	607,000 (491,000, 776,000)	2,037,000 (1,664,000, 2,592,000)
Additional treatment costs (Billion Int$)*	91.7 (71.4,107.1)	34.4 (27.6,44.1)	65.3 (53.6,83.3)	115.1 (87.9,144.5)	43.0 (33.1,55.2)	80.8 (65.5,107.1)
**Saving statins related treatment costs (Billion Int$)**	126.4 (35.7,244.1)	53.6 (16.5,110.3)	114.0 (35.7,232.2)	253.1 (81.7,489.9)	107.6 (35.8,215.1)	229.7 (71.4,458.4)
**Statin-induced diabetes (N)**	374,000 (258,000, 538,000)	161,000 (114,000, 236,000)	347,000 (244,000, 508,000)	748,000 (584,000, 965,000)	322,000 (258,000, 423,000)	694,000 (554,000, 912,000)

*, Statin prices were taken from the national centralized procurement policy bid-winning announcement file. Number aged 35–64 years old eligible for controlling LDL-C level in low, moderate, and high risk people in China was 62809000, 27579000, and 59525000 respectively. Details could be seen in the Supplementary Appendix. Int$ = 3.539 Chinese Yuan (RMB).

### Number needed to treat for ten years to prevent one ACVD event

When eligible people were treated with statins for primary prevention, the NNT_10_ decreased with the increase of ASCVD risk. The NNT_10_ was 193.0 (95%CI: 150.6–246.0), 67.3 (95%CI: 51.7–84.4), and 42.8 (95%CI: 33–53.4) for treating low to high ASCVD risk populations, respectively (Table 3). When switching low-dose to moderate-dose statins treatment, the NNT_10_ would decrease significantly in every risk groups.

### Costs

**Figure 2:**
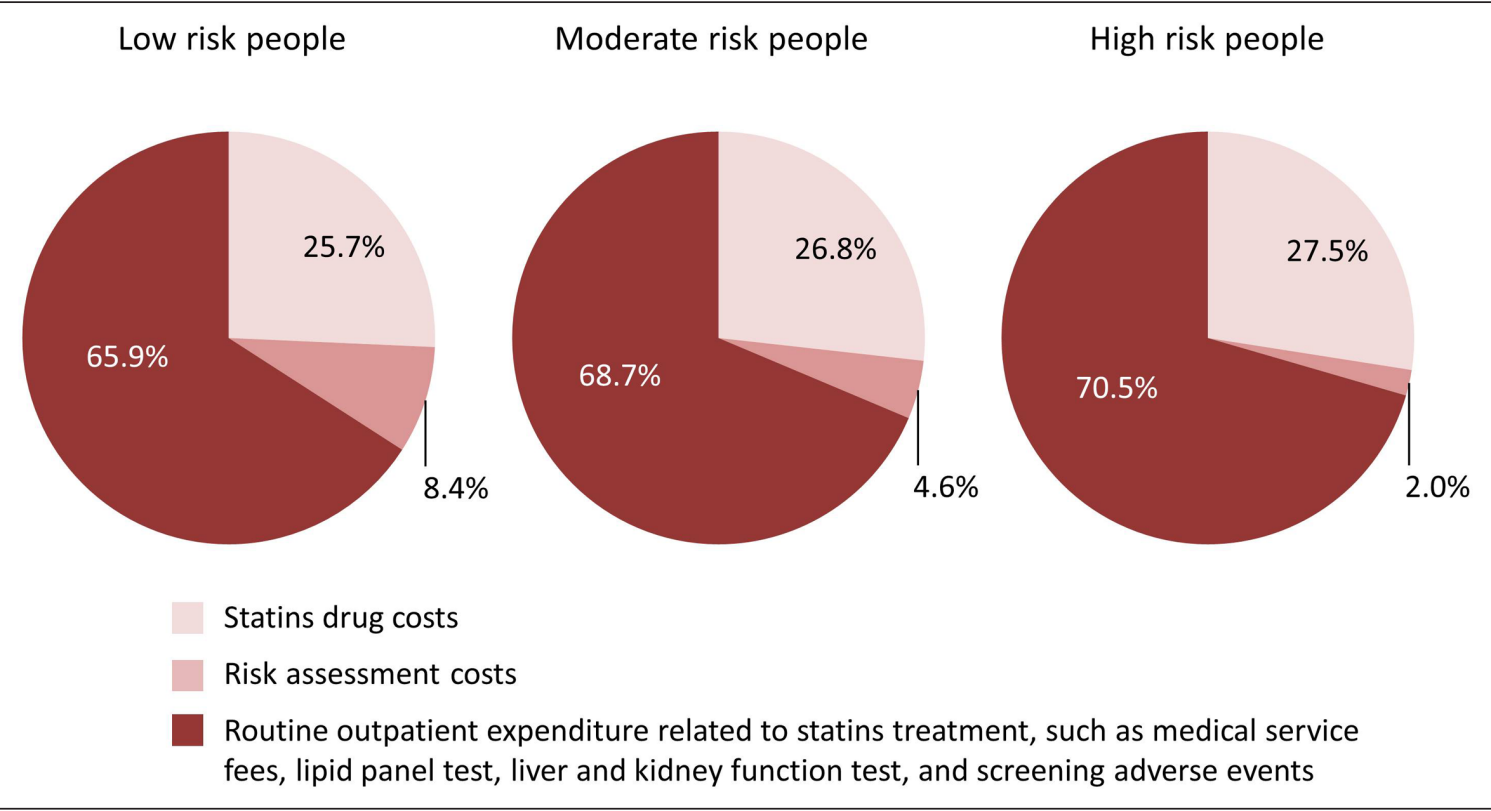
Proportion of outpatient costs of low-dose statins treatment for people without ASCVD by risk level. ASCVD, atherosclerotic cardiovascular disease. Low-dose statins: simvastatin 20 mg/day, atorvastatin 10 mg/day, or rosuvastatin 5 mg/day.

When the lower prices of statins resulting from the new centralized medicine procurement policy were used in the model, statins treatment-related costs decrease greatly compared with the pre-policy prices (Table 3 and Online Table 6). Costs for treating high ASCVD risk people with low-dose statins could decrease from cost Int$ 3,000 (95% CI: 1,500–5,300) to Int$ 1,100 (95% CI: 900–1,400) per person over 10 years from a health-care sector perspective. If eligible people aged 35–64 year old in China were all treated for 10 years for ASCVD primary prevention under the new policy, it could save 126.4 (95% CI: 35.7–244.1), 53.6 (95% CI: 16.5–110.3), and 114.0 (95% CI: 35.7–232.2) billion Int$ when treating low to high ASCVD risk populations. The results show that approximately a quarter of primary prevention costs went on statins drug costs when treating with lose-dose statins (Figure 2). The costs related to moderate-dose statins treatment were shown in Table 3 and Online Table 6.

### Cost-effectiveness

**Figure 3:**
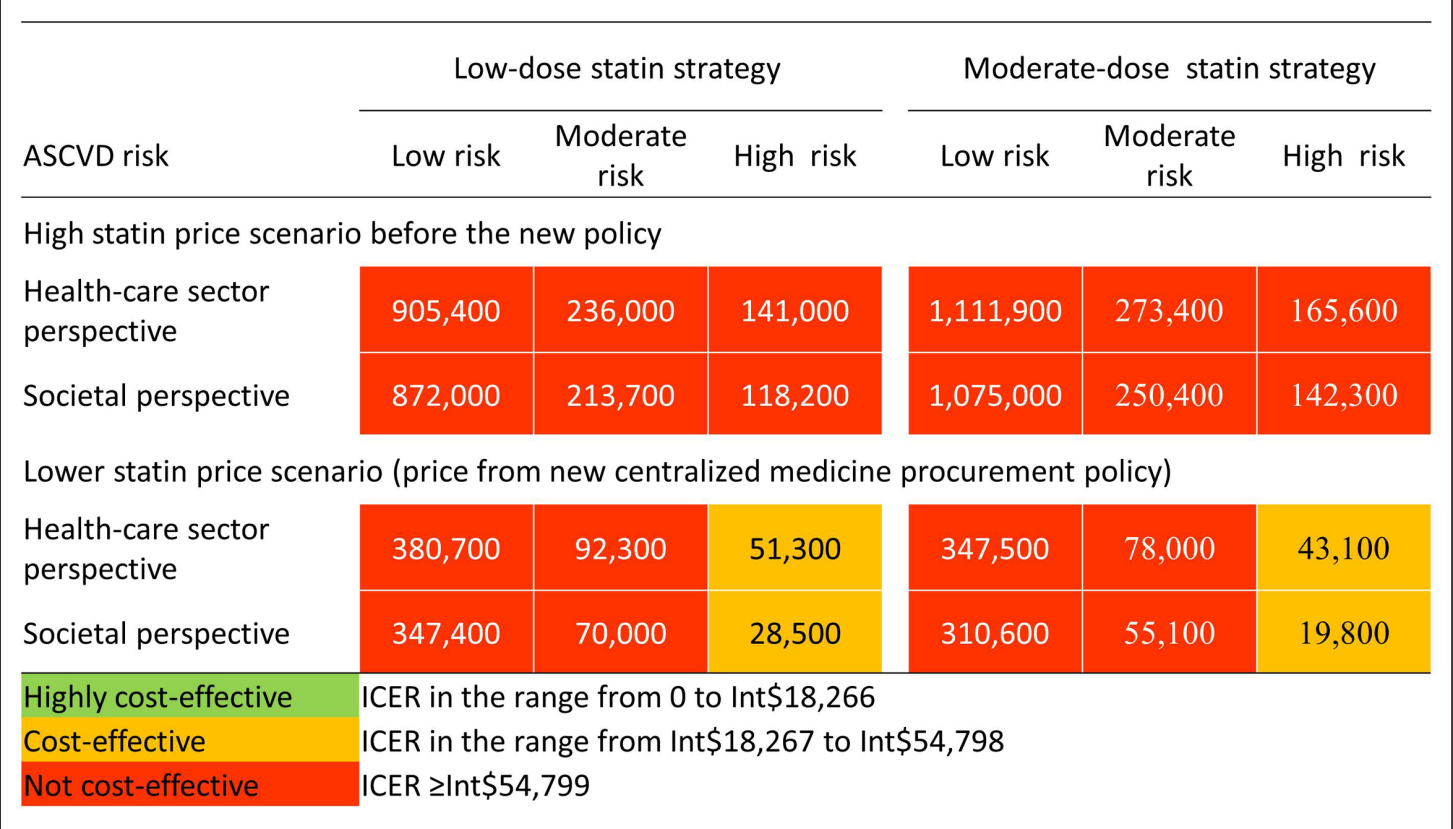
Cost-effectiveness of statin treatment among people with different risk of ASCVD. Data are incremental cost-effectiveness ratios (Int$ per QALY gained). ASCVD, atherosclerotic cardiovascular disease; ICER, incremental cost-effectiveness ratio. In healthcare sector perspective direct cost such as change of ASCVD treatment cost due to decrease of ASCVD incidence, statin medicine cost, statin treatment related cost, routine healthcare cost, and adverse events treatment cost were included. In societal perspective both direct and indirect costs were included. The indirect cost included patient-time costs, unpaid caregiver-time, transportation costs, and labor market earnings lost.

**Figure 4:**
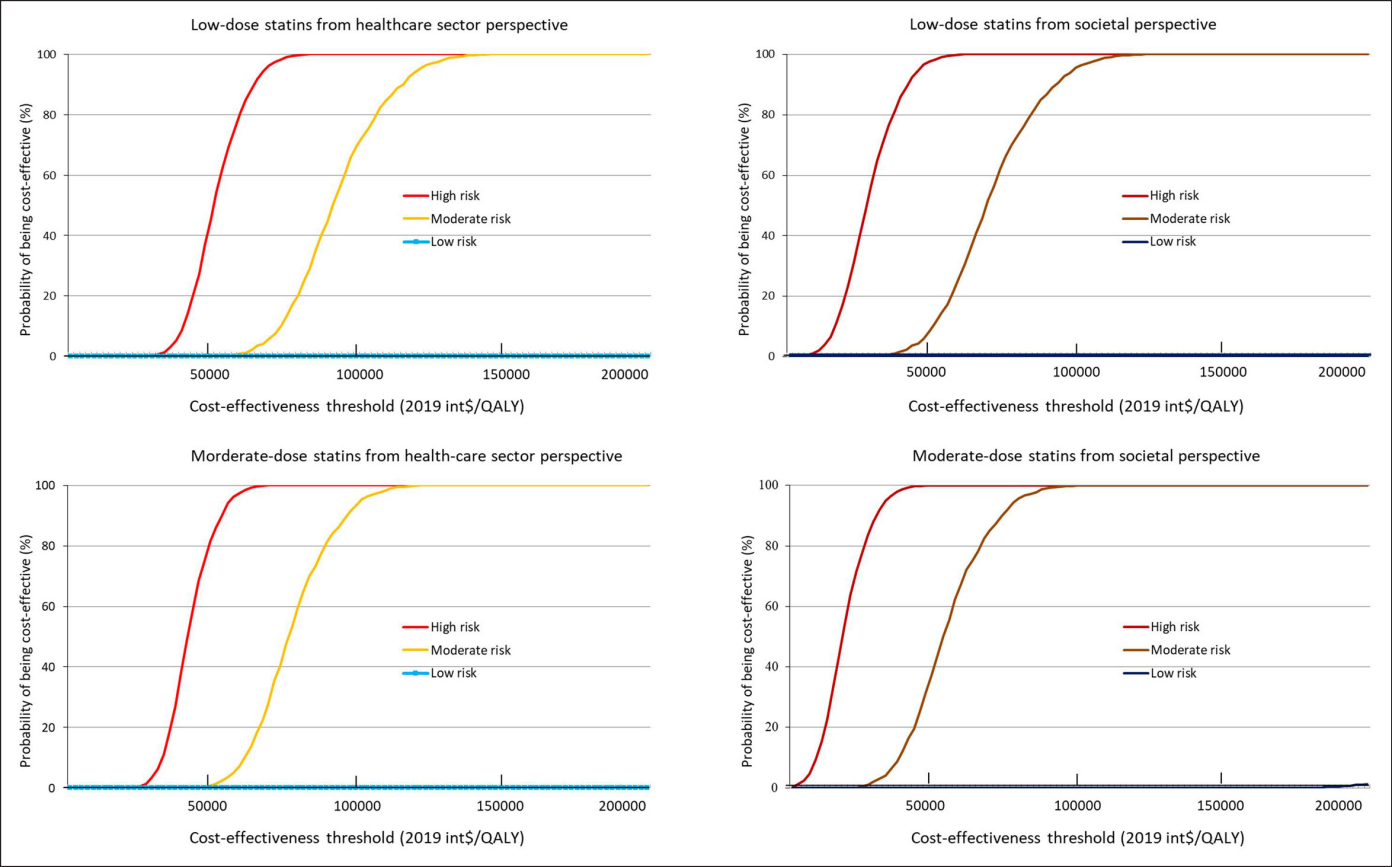
Cost-effectiveness acceptability curves for people with different 10-year ASCVD risk treated with different dose of statin.

From the health-care sector perspective, both low- and moderate-dose statin treatments were cost-effective for high ASCVD risk people when statin prices were derived from the new centralized medicine procurement policy (Figure 3, Table 3, and Online Figure 4). Monte Carlo probabilistic sensitivity analyses show treatment of high ASCVD risk people with either low- or moderate-dose statins was cost-effective in 64.9% and 90.8% of probabilistic simulations, respectively, from the health-care sector perspective. The cost-effectiveness acceptability curves are shown in Figure 4. The ICERs and probabilities of being cost-effective would be more favorable in the analysis from the societal perspective because of the saving of indirect costs (Figure 3 and Online Table 5). By contrast, for low ASCVD risk people, neither low- nor moderate-dose statins treatments were cost-effective, even when statin prices were based on the new policy (Table 3). Assuming the higher prices in place before the 2019 pricing policy, statins treatment for primary prevention would not meet standard cost-effectiveness thresholds regardless of risk level, choice of therapy, or inclusion of indirect costs (Figure 3 and Online Table 5).

### One-way sensitivity analysis

One-way sensitivity analysis results are presented in Online Table 7. When the lowest price of statins arising from the new policy was used, treating moderate risk people with moderate-dose statins could be cost-effective from the societal perspective (48,800 Int$/QALY). One-way sensitivity analyses show that the ICER was sensitive to changes to the incidence rate and the utility of statin-induced diabetes. Among low ASCVD risk people, the incidence of statin-induced diabetes might exceed the number of prevented ASCVD cases when low- or moderate-dose statin treatments were used (Table 4 and Online Figure 5).

## Discussion

Statin therapy for ASCVD primary prevention would be an effective and cost-effective intervention strategy for Chinese adults aged 35–64 years old who are ASCVD-free but at high risk of ASCVD, but only if statin prices achieved by China’s 2019 centralized medicine procurement policy are fully implemented. Moderate-dose statins treatment is more cost-effective than low-dose statins treatment. For Chinese adults with low-ASCVD risk, statin therapy is unlikely to be cost-effective even given lower drug costs.

Statin treatment simulation studies conducted in the US have shown that treating high ASCVD risk adults with statins is cost-effective, but treating low ASCVD risk people is not [6,10]. Results based on populations and health-care systems in other countries also demonstrate the value of statin treatment in high ASCVD risk populations [8,9,32]. The variation of ICERs between different countries is likely to be explained largely by the differences in statin costs and acute and chronic ASCVD treatment costs among the countries. In our study, the price of statins was the key determinant of cost-effectiveness, as in another existing study [7]. We found that implementation of the new centralized medicine procurement policy in China could decrease the average statin price by more than 80% (Table 1), which would in turn make statin treatment for primary prevention in high-ASCVD risk people cost-effective from either a health-care sector or societal perspective. In addition, we found that the annual cost of low-dose statins accounted for 27.5% and 26.8% of the total statins treatment-related costs (e.g., medical service fees for regular outpatient visits, expenses for regular biochemical measurement tests according to the guidelines, and so on) for high and moderate risk people in China (Figure 2).

A recent study estimated that statin treatment in China in people with ≥15% 10-year ASCVD risk would cost Int$ 19,600 (1 Int$ = 3.539 RMB) per QALY gained over a lifetime [33]. This lower ICER might be due to adopting a lifetime time horizon (compared with a 10-year horizon in this study), selecting a higher ASCVD risk threshold, assumption that a limited five-year statin treatment course with no additional statin treatment-related costs (i.e., routine outpatient expenditure for prescription, biochemical tests for monitoring lipids and adverse events, and adverse events treatment) would lead to persistent lifetime health, and a lower utility for chronic ischemic stroke. When we adopted the statin costs, ASCVD treatment costs, and treatment effect using data from that study in our model, and excluded the costs of risk assessment, routine prescriptions, lipid panel test, and medication-related adverse event monitoring and treatment, the ICER decreased by 60% compared with our main analysis estimate.

The new centralized medicine procurement policy reduced the average price of the 25 selected medicines by 52% in the 11 pilot cities and the maximum reduction could reach 93% [34]. Additionally, the usage rate of the selected medicines increased from 50% to 90% in the pilot cities [34]. In December 2019, China’s Central Government decided to extend the centralized medicine procurement nationwide in 2020. At least 176.0 million tablets of low-dose simvastatin (20 mg/tablet) and 8.8 million tablets of moderate-dose simvastatin (40 mg/tablet) will be purchased in 31 provinces (autonomous regions, municipalities) [35].

In China, each city had a unique reimbursement policy for some chronic diseases (such as cancer, cardiovascular disease, and diabetes, etc.), with both high reimbursement rates and high reimbursement cap lines. Outpatient treatment cost for patients only with dyslipidemia would not be covered by above-mentioned policy in the resident medical insurance scheme. Patients only with dyslipidemia had to use the coordination policy in which the median of the reimbursement cap line in 45 cities was approximately 85 Int$ (interquartile range: 34–226 Int$), so the on-going new centralized medicine procurement policy decreased the out-of-pocket costs of people who need statins treatment for primary prevention of ASCVD (Online Table 8).

### Limitations

The current study was constrained by the baseline age range of the CMCS study and therefore did not include people older than 64 years old. Older individuals comprise a growing portion of the Chinese population and future research should strive to include them. CMCS baseline cholesterol and other risk factor levels observed in 1990s and may be lower than the current levels representative of Chinese adults. The CMCS cohort may not be representative of contemporary Chinese adult population. Comparing to the risk factor level in 2012 nation-wide survey and published in the Report on Chinese Residents’ Chronic Diseases and Nutrition [36], the average level of diastolic blood pressure, TC, and fasting blood glucose of the CMCS population used in this study were similar to those in the national survey (Online Table 9), but the average systolic blood pressure was lower and HDL-C was higher than those in the national survey. Past randomized, controlled clinical trials that established the effect of statins on lowering ASCVD risk did not enroll many Chinese participants. We applied the average effects estimated in clinical trials meta-analyses of these trials, which may not exactly be generalizable to China [18]. Future research should explore the effect of statin use on LDL-C and ASCVD risk in more diverse populations, including Chinese adults.

Higher statin prices reflecting typical prices seen before the 2019 centralized procurement policy were obtained from the website that indicates statin prices for hospitals located in Beijing [13]. The national representativeness of these statin prices is not known, as the Chinese government has continued trying to lower prices and every province has its own project and bidding system [37]. To calculate indirect costs, we used an average wage estimated based on statistical data from 31 provinces (autonomous regions, municipalities) of China for the simulation population, meaning that our results do not reflect the effect of income diversity on the results.

### Conclusion

By lowering statin prices substantially, China’s new centralized medicine procurement policy will improve the cost-effectiveness of statins for primary ASCVD prevention in adults aged 35–64 years. After 10 years of treatment, new lower prices make statin treatment cost-effective among high ASCVD risk people from both a health-care sector and a societal perspective.

## Data Accessibility Statement

All parameters used in this study are listed in the manuscript or supplementary file. Readers who are interested in the individual risk factor levels of the Multi-provincial Cohort Study used to calculate the 10-year ASCVD risk in this study can contact Dr. Dong Zhao by e-mail.

## Additional File

The additional file for this article can be found as follows:

10.5334/gh.830.s1 Supplementary Material. The additional information of methodology, online figures, and online tables.
