# Supplementary material for: Impact of China’s Low Centralized Medicine Procurement Prices on the Cost-Effectiveness of Statins for the Primary Prevention of Atherosclerotic Cardiovascular Disease
Source: Glob Heart. 2020 Jun 25;15(1):43. doi: 10.5334/gh.830 (PMC7427664; doi:10.5334/gh.830)
Supplement: Supplementary Material. — The additional information of methodology, online figures, and online tables. [file gh-15-1-830-s1.pdf]

Supplemental Material

Contents

Supplemental Methods 1

Model design 1

Simulation Cohort.. 2

Risk stratification.. 4

Costs estimation.. 4

Adverse events.. 7

Utilities 8

Model Calibration and Validation.. 8

Estimating effect of statin price change on outpatient treatment cost for ASCVD in chronic stage when the price of statin decrease.......... 9

Methods for estimating the effect of statins treatment implementing in whole China.. 11

Online Figures..13

ONLINE FIGURE 1. China statin decision ASCVD model structure 13

ONLINE FIGURE 2. Health status and transition of the simulation model before and after intervention 15

ONLINE FIGURE 3. Ratio of observed to predicted rate of ASCVD incidence, mortality, and non-ASCVD mortality rate (%) by gender after model calibration.. 16

ONLINE FIGURE 4. Cost-effectiveness of statin treatment among people with different risk of ASCVD. Data are incremental cost-effectiveness ratios (Chinese Yuan (RMB) per QALY gained) 17

ONLINE FIGURE 5. Prevented ASCVD number and incidence of statin induced-adverse events after implementing the statin intervention strategy for 10 years.. 18

Online Tables 20

ONLINE TABLE 1. Baseline characteristics of participants in Chinese Multi-provincial Cohort Study 20

ONLINE TABLE 2. Indirect costs for model input........................ 21

ONLINE TABLE 3. Equations for estimating indirect costs in the model 22

ONLINE TABLE 4. Model input parameters of utility and time .. 23

ONLINE TABLE 5. Effectiveness and cost-effectiveness of 10-year statin treatment among Chinese adults stratified by ASCVD risk in Chinese Multi-provincial Cohort Study population.......................... 24

ONLINE TABLE 6. Mean 10-year incremental health-care cost (Int $) of statin vs. no statin treatment for each eligible person stratified by ASCVD risk... 27

ONLINE TABLE 7. Results of one-way sensitivity analysis base on the statin price from the new centralized medicine procurement policy and societal perspective... 28

ONLINE TABLE 8. Reimbursement policy for outpatient service in the urban and rural resident medical insurance scheme in selected cities of China... 30

ONLINE TABLE 9. Risk factor level in recent national survey and baseline survey of CMCS participants 37

Supplemental Methods

Model design

An atherosclerotic cardiovascular disease (ASCVD) microsimulation model was build based on a decision tree analytic model and the data inputs of ASCVD risk, probabilities of ASCVD incidence, and transitions between disease states were mainly derived from the long-time follow-up Chinese Multi-provincial Cohort Study (CMCS) [1]. The computer simulation model was designed to explore a range of statin effects and costs as part of primary ASCVD prevention strategies. Health states, accompanied by corresponding health-related quality of life and costs in this microsimulation model were referred to the design of the Cardiovascular Disease (CVD) Policy Model-China, an established computer-simulation, state-transition model of incidence, prevalence, mortality, and costs of CVD in China [2]. In this study, ASCVD comprised acute coronary event (acut coronary events (ACE), including myocardial infarction, cardiac arrest, and coronary death [International Classification of Diseases, 10th Revision (ICD-10) codes: I20-I25 and I46] and ischemic stroke (ICD-10 codes: I63 and I69.3).

According to the design of this microsimulation model, people without history of ASCVD would develop ASCVD under the effect of risk factors of ASCVD. The courses of ASCVD were divided into acute ASCVD stage (first 30 days after an acute event), chronic ASCVD stage (after the first 30 days), recurrent stage, and death. Each stage was assigned corresponding transition probabilities, quality-adjusted life-year (QALY) weights, costs, and duration of the stage. Since the QALY weights and medical costs were all different for acute coronary events and ischemic stroke, the model was constructed by type of ASCVD accordingly. As a result, there are four stages and 16 ASCVD states in the microsimulation model (**Online Figure 1**). The four health stages comprise healthy/without ASCVD, acute ASCVD (first 30 days after an acute event), chronic ASCVD (after the first 30 days), and death. There are four acute ASCVD health states (acute coronary events out-of -hospital death, in-hospital acute coronary events death, out-of-hospital ischemic stroke death, and in-hospital ischemic stroke death) and 12 states after acute stage of ASCVD (chronic stage after suffered from acute coronary events, recurrent acute coronary events, acute coronary events followed by first ischemic stroke, chronic acute coronary events death, chronic acute coronary events followed by ischemic stroke death, chronic acute coronary events followed by non-ASCVD death, chronic ischemic stroke, recurrent ischemic stroke, ischemic stroke with onset of first acute coronary events, chronic ischemic stroke death, chronic ischemic stroke

followed by acute coronary events, and chronic ischemic stroke followed by non-ASCVD death). Each health state is accompanied by corresponding health-care cost, indirect cost loss and quality of life.

Simulation Cohort

The simulation cohort was derived from the CMCS study, a nationwide, multi-center cohort study on the determinants of cardiovascular disease (CVD) in China. Informed consent was obtained from all participants, who were all free of cardiovascular disease at the time of enrollment. Participants were selected using a multistage sampling method. First, centers in each region were selected non-randomly in China. Next, a stratified random sampling for each sex and 10-year age group was performed in each center for the baseline survey [3]. The median follow up time was 14.2 years in 2014. The participants were followed up every two or four years to get the information for ascertaining new ASCVD events and all-cause death. The overall participation rate at last follow-up was 82.1% in 2014.

The baseline risk factors of ASCVD of the cohort people were used to calculate the individual predicted 10-year ASCVD risk which was the personal cumulative incidence probability and used to count the incidence number of ASCVD. The individual 10-year ASCVD risk of simulated individuals used in this study was calculated using a China-specific 10-year first ASCVD risk assessment equation [4]. The observed sex and 10-year age specific proportion of acute coronary events among ASCVD, proportion of patients die in acute stage of each type of ASCVD after first onset, and proportion of patients suffered from the recurrent ASCVD were used to estimate the population number in each stage of ASCVD (**Table S1**).

Table S1. Probability of the transition of ASCVD after onset in ten years in the model

	Male			Female		
Category	35-44 age	45-54 age	55-64 age	35-44 age	45-54 age	55-64 age
Proportion of acute coronary events in the total ASCVD incidence (%)	53.1	39.1	38.6	28.6	35	33.8
Case-fatality rate of acute coronary events in acute stage (%)	27.9	27.9	22.7	21.4	11.4	20.0

Case-fatality rate of acute coronary events after acute stage of first onset (%)	2.3	1.5	10.9	7.1	2.9	4.4
Proportion of non-ASCVD death among all deaths with history of acute coronary events (%)	4.7	1.5	13.6	7.0	2.9	4.5
Case-fatality rate of ischemic stroke in acute stage (%)	10.5	3.8	9.7	2.9	6.2	6.8
Case-fatality rate of ischemic stroke events after acute stage of first onset (%)	5.3	7.5	13.1	8.6	3.1	11.4
Proportion of non-ASCVD death among all deaths with history of ischemic stroke (%)	5.3	13.2	15.4	5.7	9.2	10.2
Non-ASCVD death rate (%)	1.9	2.8	8.8	1.4	3.1	6.4
Recurrent rate of acute coronary events (%)	14.0	10.3	3.6	7.1	5.7	4.4
Recurrent rate of ischemic stroke (%)	7.9	8.5	15.4	8.6	9.2	8.0
Incidence rate of ischemic stroke among people with history of acute coronary events (%)	9.3	2.9	6.4	0	8.6	8.9
Incidence rate of acute coronary events among people with history of ischemic stroke (%)	2.6	0.9	1.7	0	4.6	1.1
Proportion of out-hospital death among death from acute coronary events (%) *	70.6	70	68.3	75.8	73.9	64.9
Proportion of out-hospital death among death from ischemic stroke (%)*	41.5	45.8	58.6	57.8	59.3	64.1

ASCVD, atherosclerotic cardiovascular disease

*, Data from the surveillance study of thrombotic diseases in Beijing (unpublished). All other data were from the Chinese Multi-provincial Cohort Study

Risk stratification

High risk of ASCVD was determined by presence of one of the following conditions which recommended by Chinese Guidelines for the Management of Dyslipidemia in Adults:[5] 1) LDL-C ≥ 4.9 mmol/L or TC ≥ 7.2 mmol/L; 2) diabetes and LDL-C in the range of 1.8-4.8 mmol/L (or TC 3.1-7.2 mmol/L) and age≥40 years; 3) 10-year ASCVD risk ≥10%. Other people were divided into moderate (10-year ASCVD risk ≥5% and <10%) and low (10-year ASCVD risk <5%) risk categories according to the 10-year ASCVD risk. People who were classified as high risk defining by specific characteristics, such as LDL-C ≥ 4.9 mmol/L, their risk were aslo calculated by using the 10-year ASCVD risk assessment equation for Chinese [4].

Costs estimation

Costs in this study were estimated both from the societal perspective (both direct and indirect costs were included) and health-care sector perspective. Parameters were listed in **Table 1** in the manuscript and **Online Table 2**). The direct cost included per capita total expenditure on health, ambulance and out-of-hospital treatment costs for each type of ASCVD (acute coronary event or ischemic stroke), hospitalization expense, post-event outpatient ASCVD management cost, risk assessment cost, daily statin medication expenditure, lipid panel test cost, adverse event monitoring cost, and adverse event treatment cost. The indirect costs would incur after the onset of ASCVD or death which included patient-time costs, unpaid caregiver-time costs, transportation costs, labor market earnings lost, and cost of unpaid lost productivity due to the disease [6].

The indirect cost included financial losses for the patients and their caregivers (**Online Table 2**). The patient-time cost due to hospitalization was calculated by multiplying the daily wages by average duration of hospitalization and employment rate (**Online Table 3**). The patient-time cost due to outpatient clinic visit was calculated by multiplying the daily wages by time consumption for each visit, visit time per year, survival year after onset of ASCVD, and employment rate. Unpaid caregiver-time cost due to hospitalization was calculated by multiplying the daily wages by average duration of hospitalization and employment rate (It was supposed every hospitalized ASCVD patient need one caregiver). Unpaid caregiver-time cost due to outpatient clinic visit was calculated by multiplying the daily wages by time consumption for each visit, visit time per year, survival year after onset of ASCVD, disability rate, and employment rate (It was supposed every disabled ASCVD patient need one

caregiver to accompany for visiting outpatient clinic. Other patients can go to hospital alone). The disability of ASCVD patients was defined as patients with acute coronary events suffered from heart failure or ischemic stroke patients whose modified Ranking Score (mRS) ≥2.[7,8] Transportation costs were calculated by multiplying trip cost by trip time per year and survival year after onset of ASCVD. Labor market earnings lost for each patient included cost loss due to disability and cost loss due to premature death. The cost loss due to disability was calculated by multiplying average yearly salaries by survival year after onset of ASCVD, disability rate, and employment rate. The cost loss due to premature death was calculated by multiplying average yearly salaries by life year loss due to ASCVD death, and employment rate. Cost of unpaid lost productivity due to illness (uncompensated household production) for each patient included additional cost due to disability and additional cost due to premature death. The average yearly wage was estimated based on the average paid salaries for people who either worked in the non-private and private institute in the 31 provinces (autonomous regions, municipalities) of China after weighting by the employment figure.

The employment rate used in the study was calculated base on the unemployment rate which was from data of International Labour Organization [9]. The unemployment comprised 1) without work (paid employment or self-employment), 2) currently available for work, or 3) seeking work. Parameters for estimating indirect cost and their resource were listed in **Table S2**.

Table S2. Parameters for indirect cost estimation

Category	Parameters	Data resource
**Duration of hospitalization (Day)**		
Acute coronary events	8.9	China's Health and Family Planning Statistical Yearbook
Ischemic stroke	10.9	
Rhabdomyolysis	8.4	
**Average yearly salaries (Int$ /year)**	17,295.1*	China Statistical Yearbook 2018
**Average yearly salaries for household service (Int$ /year)**	12,981.3*	
**Daily wages of caregiver for hospital visit (Int$ /day)**	66.3*	

**Transportation costs (Int$ /single trip)**	3.0*	
**Unemployment rate (%)**	3.8	Website of International Labour Organization (China's profile)
**Disability rate (%)**		
Acute coronary events	6.6	Improving Care for Cardiovascular Disease in China-Acute Coronary Syndrome Project
Ischemic stroke	36.2	Prospective Cohort Study on the Incidence and Outcome of Patients with Post-stroke Depression in China

*, Costs were inflated to 2019 int$.

The estimation of variance of costs

To get the distribution of costs for Monte Carlo probabilistic sensitivity analyses, the 95% confidence interval of each type of cost were calculated base on the mean and standard error (SE). The SE was calculated by deviding standard deviation by the square root of number of sample size. For annual per capita total expenditure on health, the China' Health Statistics Yearbook reported the national average and province-specific average expidenture (N=31) [10]. The province-specific expidenture was used to calculate the SE of annual per capita total expenditure. For hospitalization expense of myocardial infarction and ischemic stroke, the statistic yearbook reported the national and five level of hospital's averge hospitalization expense. The level-specific expenses then were used to estimate the SE. Limited by the data accessibility, the SEs of other outpatients expenditure used in this study were estimated base on the ratio of SE to mean of annual per capita total expenditure.

The mean and standard deviation of yearly statin treatment cost, which used in the Monte Carlo probabilistic sensitivity analysis, were estimated based on the price for each 1 mg statin. The prices and package information of statin (N=49) before the new policy were from the Integrated Management Platform of Beijing Medicine Sunshine Purchase database [11]. The lower statin prices (two types of statin) after the execution of the new policy was from the bidding result announcement [12]. Because the indirect costs were estimated base on the annual income, the the 95% confidence interval of each type of indirect cost were calculated base on the ratio of SE to mean of national annual income, and the SE was calculated base on the province-specific average income.

Adverse events

Adverse events related to statin treatment were accounted for only if they met the following criteria: the adverse event changes the quality of life, it incurs additional expenditure for treatment, or it results in stopping treatment. After synthesizing the information from meta-analyses,[13-15] clinical trials,[16] Chinese guidelines for the management of dyslipidemia,[5] and Chinese expert consensus for statin safety,[17] the myopathy (unexplained muscle symptoms with a creatine kinase (CK) >10x upper limit of normal [ULN]), rhabdomyolysis (A subset of myopathy in which there is evidence of end-organ damage (e.g. doubling of serum creatinine compared to value at baseline) and significant muscle damage (CK >40x ULN)), Elevated liver enzymes (Alanine aminotransferase (ALT) >3x ULN and bilirubin ≥2x ULN), and diabetes (Plasma glucose ≥11.1 mmol/L if fasted <8 h or ≥7.0 mmol/L if fasted ≥8 h, or baseline HbA1c ≥48 mmol/mol, or use of hypoglycaemic medication) were included in this study as adverse events caused by statins treatment.[16]

For people suffered from the myopathy, the experts consensus suggested stop taking statins or switch to other type of statins and do proper amount exercise.[17] People suffered from the rhabdomyolysis should stop statins treatment and received intravenous rehydration therapy if necessary. If people get diabetes during statins treatment, the expert consensus suggested patients should control weight, intervene their lifestyle and dietary habits, and receive hypoglycemic agent. For people whose ALT level was more than 3 times of ULN should stop taking statins and need not receive additional treatment. So in this study, the onset of myopathy was supposed to have effect on QALY and cost of rechecking biochemical panel for monitoring the recovery. Elevated liver enzymes were assumed not to be clinically meaningful but resulting in drug withdrawal (no adherence). Finally, diabetes, rhabdomyolysis, and myopathy were included in the simulation.

The incidence rates of adverse events were from the Chinese participants who were enrolled in the Treatment of HDL to Reduce the Incidence of Vascular Events (HPS2-THRIVE) study.[16] The primary aim of HPS2-THRIVE study was to assess the effect of addition of extended-release niacin 2 g plus laropiprant 40 mg daily to simvastatin 40 mg daily on the incidence of major vascular events. In this study 10932 Chinese were randomly allocated to the two treatment groups and followed for a median of 3.9 years. The incidence rates of adverse event observed in the group treated with simvastatin and placebo were used in this study.

Utilities

Every health state was assigned a utility, which was used to estimate the benefits and harms of statin use on health in the model (**Online Table 4**). Chronic ASCVD utilities were obtained from a systematic review of health-related quality of life studies using EuroQOL 5 Dimension (EQ-5D) questionnaire estimates in Chinese adults [18]. A value of 0.5 was assigned to the acute stage of ASCVD (based on clinical judgment) [19,20]. The utility of statin-related adverse effects was obtained from the Global Burden of Disease (GBD) 2016 study [21].

Model Calibration and Validation

The model was calibrated to achieve the best fit (minimal residual difference) between the simulation model's predicted rate and observed rate. The model outputs, person-year incidence of ASCVD (separated by acute coronary events and ischemic stroke) and non-ASCVD mortality, were calibrated according to the observed yearly incidence or mortality. After calibration of incidence of ASCVD, the ratio of ASCVD deaths to ASCVD incidence (case fatality) from the model output were calibrated according to the observed yearly case-fatality rate. The age-sex-specific observed rates for model calibration and their resource are listed in **Table S3**. The incidence rate of ASCVD was calibrated by internal validation to make the risk factor levels, statin treatment rate, and incidence rate of ASCVD matched each other. The ASCVD case-fatality rate and non-ASCVD mortality rate were calibrated by external validation to make them match the contemporaneous health-care costs used in the model, ASCVD treatment related costs, ASCVD case-fatality rate, and non-ASCVD mortality rate match each other.

Table S3. Parameters for model calibration

	Incidence rate * (1/100000)		Case-fatality rate (%)†		All-cause death‡ (1/100000)		
Age	Acute coronary events	Ischemic stroke	Acute coronary events	Ischemic stroke	City	Village	All §
**Male**							
35 - 39	76.5	76.4	26.3	1.8	93.7	148.0	116.8

40 - 44	142.7	126.4	23.9	5.0	172.7	228.6	196.5
45 - 49	111.1	202.3	24.5	5.4	245.5	343.0	287.1
50 - 54	239.7	360.8	28.7	6.0	621.8	742.7	673.4
55 - 59	308.2	516.2	33.5	7.6	643.7	700.9	668.1
60 - 64	555.3	970.4	39.2	9.4	1382.7	1403.2	1391.4
**Female**							
35 - 39	18.9	49.3	50.0	2.4	40.1	57.7	47.6
40 - 44	44.6	109.3	52.3	6.1	73.2	89.8	80.2
45 - 49	81.1	146.5	40.5	4.1	111.0	143.4	124.8
50 - 54	116.2	228.0	37.3	5.0	277.0	348.8	307.6
55 - 59	256.4	416.6	37.5	7.1	253.8	316.2	280.4
60 - 64	169.1	607.3	38.2	8.4	660.4	725.7	688.3

*, Data were from the Chinese Multi-provincial Cohort Study.
†, Data were from the surveillance study of thrombotic diseases in Beijing (unpublished).
‡, Data were from China's Health and Family Planning Statistical Yearbook.
§, Estimated base on the regional-specific mortality rate and its population weight which were used for model calibration.

During calibration the correction coefficient was the slope which was achieved through fitting linear regression equation without intercept using the sex and 5-year age-specific observed rates and model predicted outputs [22]. The observed rate was set as dependent variable. The calibrated probability of ASCVD incidence, ASCVD case-fatality rate, and non-ASCVD mortality for each simulated individual in the cohort were obtained by multiplying by the slope and the pre-calibrated probability. The ratios between calibrated simulated rate and observed rate were all near one and shown in **Online Figure 3**.

Estimating effect of statin price change on outpatient treatment cost for ASCVD in chronic stage when the price of statin decrease

When we explored the effect of the decreased statin price achievable through the national centralized medicine procurement policy on the cost-effectiveness of statin treatment in primary prevention, the cost of statin as part of outpatient treatment expenditure for patients with ASCVD would also decrease, which would affect cost-effectiveness by lowering annual treatment cost of patients in the chronic stage of ASCVD (that is, secondary prevention) since statins were recommended for long-term use for ASCVD patients. The drug expenditure of

statin for ASCVD patient in chronic stage was estimated based on the annual drug expenditure of ASCVD patient in chronic stage. Drug expenditure was calculated by multiplying the annual treatment cost of ASCVD in the chronic stage by the percentage of drug expenditure expected as part of the annual treatment cost of ASCVD. Since the data related to proportion of statin expenditure among the drug expenditure in chronic stage of ASCVD was not specifically quantified, the statin cost was estimated by multiplying the total expected drug expenditure for chronic ASCVD patients by the proportion of annual drug cost of statins among the four types of recommended drugs (aspirin, statins, β-blockers, and angiotensin-converting enzyme inhibitors, **Table S4**). The statin usage rate of ASCVD patients in chornic stage was from large sample size investigations for secondary prevention of cardiovascular disease [23,24]. Overall, the expenditure on statins for chronic ASCVD patients was estimated by multiplying the annual treatment cost of chronic ASCVD by the percentage of drug expenditure, proportion of the statin cost among the four drugs, and the statin usage rate (**Table S5**).

Table S4. Estimation of the proportion of statins cost among the yearly drug expenditure

Medicines for secondary prevention		Daily dose for secondary prevention (mg/day)*	Proportion of yearly cost among the four drugs (%)#
Aspirin		150	3.5
ACE-inhibitor	Captopril	150	18.6
	Lisinopril	10	
β blocker	Bisoprolol	10	32.4
	Metoprolol	200	
	Atenolol	100	
Statins	Simvastatin	40	45.5
	Atorvastatin	20	
	Rosuvastatin	10	
Sum			100

* , The daily doses were from the recommendations of Chinese guidelines for the prevention of cardiovascular diseases(2017) [25], expert consensus document on appropriate use of beta-adrenergic receptor blocker in patients with cardiovascular diseases [26], and Chinese expert consensus document on angiotensin converting enzyme inhibitors in cardiovascular diseases [27].

#, Except aspirin, all prices of drug were from the series of documents about the maximum retail price of essential medicines issued by the Chinese National Development and Reform Commission from 2009 to 2012. The prices of aspirin was from two websites of online pharmacy (https://www.yaofangwang.com; https://www.111.com.cn).

Table S5. Parameters for estimation of statin cost in chronic stage

	Statin utilization rate		Percentage of drug expenditure (%)	Proportion of statins cost among the drug expenditure (%, from table 5)
	Rate (%)	Data collection year		
Coronary heart disease	34.6*	2010	51.3†	45.5
Ischemic stroke	10.9‡	2012	50.3†	45.5

*, Resource: ER Atkins, Du X, Y Wu, R Gao, A Patel, CK Chow. Use of cardiovascular prevention treatments after acute coronary syndrome in China and associated factors. INT J CARDIOL 2017;241:444-9.
†,Resource: National Health and Family Planning Commission of China. China Health and Family Planning Yearbook
‡, Resource: W Longde, Y Ling, H Yang, Z Yi, W Yongjun, J Xunming, N Xiaoyuan, Q Qiumin, H Li, X Yuming, L Mei, S Jiayi, L Jing, Z Dong. Fixed-dose combination treatment after stroke for secondary prevention in China: a national community-based study. STROKE 2015;46:1295-300.

Methods for estimating the effect of statins treatment implementing in whole China

In this study, the statins treatment effect on preventing ASCVD and budget impact of statins price change due to the new medicine price policy when all eligible people aged 35-64 years old in China were to receive statins for primary prevention were also estimated. For estimating the treatment effect of improving statins use among all eligible 35-64 years old Chinese with different ASCVD risk, we first figured out the prevented ASCVD number for treating each eligible person in the microsimulation model stratifying by ASCVD risk level both in main and probabilistic analysis. Then, estimated the prevented number in the whole targeted population by multiply the prevented number per eligible person by the total eligible population number in the ASCVD risk category in China.

For estimating the budget impact due to the new policy, first we estimated the 10-year statins treatment costs for treating each eligible person in the microsimulation model both base on statin prices before the execution of the new centralized medicine procurement policy and lower price from the new policy in each ASCVD risk category. Then use the cost difference per person estimated base on the two types of price and total eligible for treatment number to illustrate the budget impact of statins price change for China's ASCVD primary prevention.

National population number eligible for treatment

To evaluate the effect of fully implemention of statin treatment in primary prevention of ASCVD aged 35-64 years old Chinese with the low-price statins from the new centralized medicine procurement policy on the change of statins related treatment costs for primary prevention, and the prevented incident number of ASCVD, national population number eligible for treatment was need to be estimated. The national number of eligible for treatment aged 35-64 years old and without ASCVD was calculated based on the population number, proportion of each 10-year risk category, percentage of eligible for treatment, and prevalence rate of ASCVD (**Table S6**).

Table S6. Age-specific population number free of ASCVD

Age group (Year)	Population number (N)	Prevalence rate (%)		Population number free of ASCVD (N)
		Stroke	Ischemic heart disease	
35-39	96,158,901	0.62	0.38	95,190,072
40-44	113,178,017	0.99	0.76	111,201,745
45-49	124,997,611	1.73	1.23	121,286,098
50-54	116,616,487	2.90	1.82	111,115,044
55-59	71,252,091	4.55	2.59	66,166,842
60-64	80,879,331	6.45	3.63	72,732,014

ASCVD, atherosclerotic cardiovascular disease

The population number was from China's Health Statistics Yearbook in which the age-specific population number in 2016 was reported [10]. The age-specific prevalence rate of ASCVD were from the website of Global Burden of Disease Collaborative Network (Available from http://ghdx.healthdata.org/gbd-results-tool) [28]. The proportion of each 10-year risk category and percentage of eligible for treatment were from CMCS study (Table 2 in the main manuscript). The formula was: national eligible population number= population number × (1-prevalence rate of ASCVD) × proportion of each 10-year risk category × percentage of eligible for treatment.

Online Figures

ONLINE FIGURE 1. China statin decision ASCVD model structure

a) Interventions

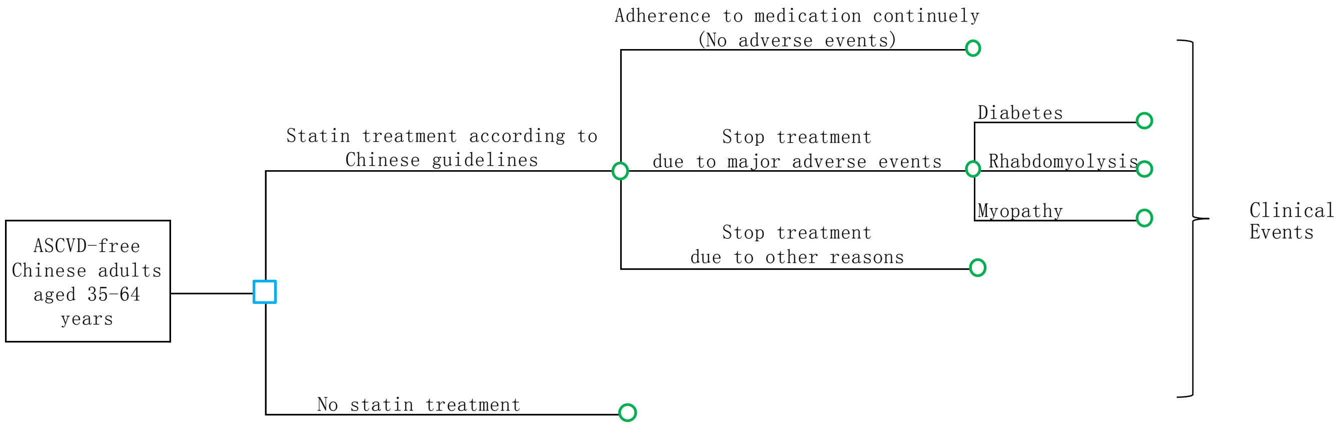

b) Clinical Events

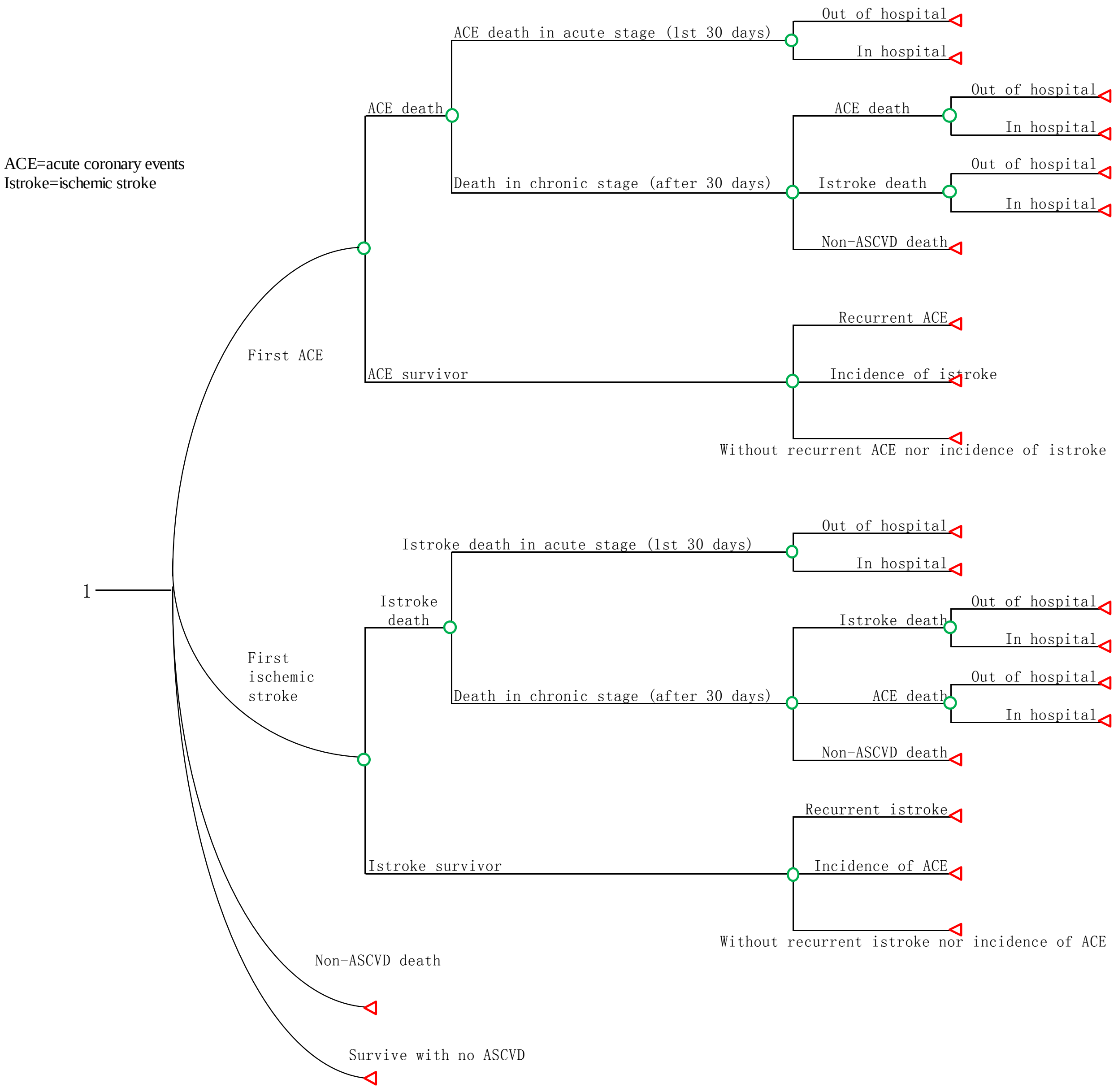

ACE, acute coronary events; Istroke, ischemic stroke; Non-ASCVD, non-atherosclerotic cardiovascular disease

ONLINE FIGURE 2. Health status and transition of the simulation model before and after intervention

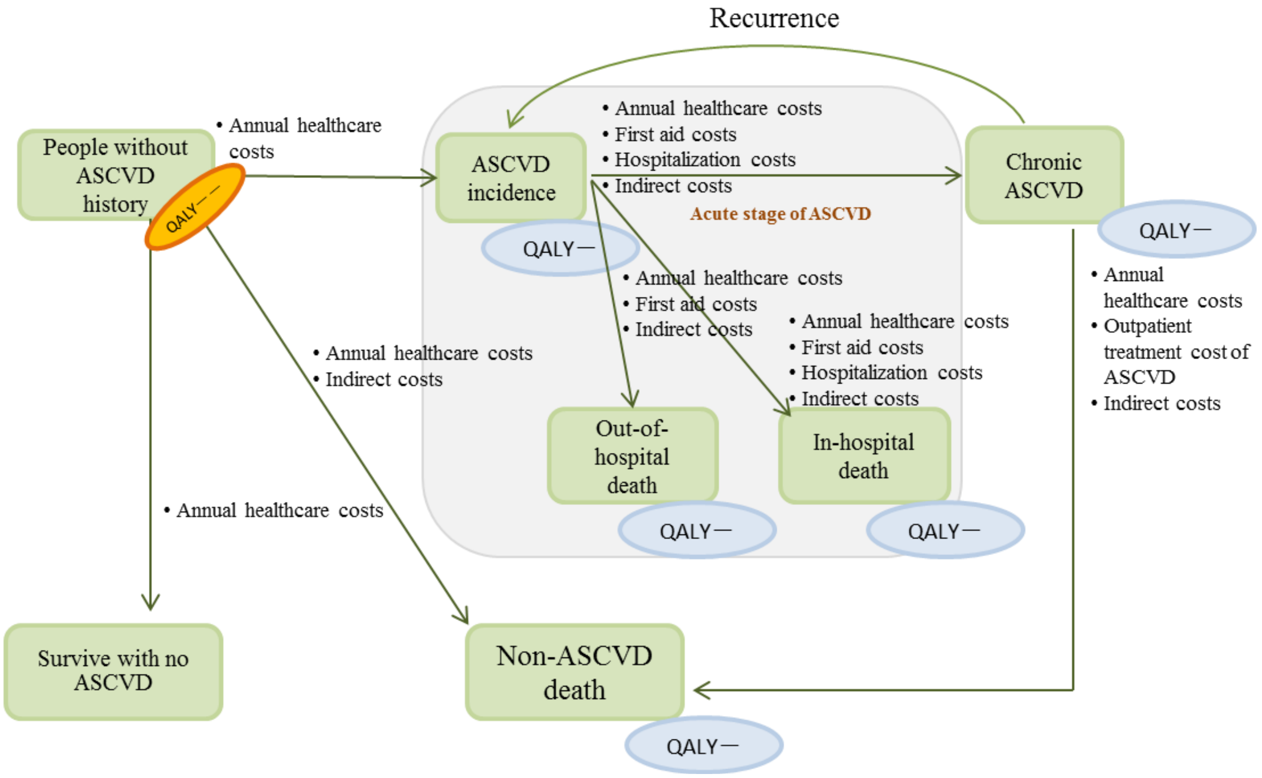

When with statin treatment, part of people in the status of “ASCVD incidence” would be moved to the status of “survive with no ASCVD” due to the treatment, meanwhile the model would include statin intervention costs (costs of risk assessment, daily statin medications, registered fee, lipid panel test, adverse events monitoring), costs and QALYs loss due to the onset of adverse events.

ASCVD, atherosclerotic cardiovascular disease, including acute coronary event and ischemic stroke; QALY—, quality-adjusted life-year loss due to the onset of ASCVD; QALY——, QALY loss due to the onset of adverse events.

ONLINE FIGURE 3. Ratio of observed to predicted rate of ASCVD incidence, mortality, and non-ASCVD mortality rate (%) by gender after model calibration

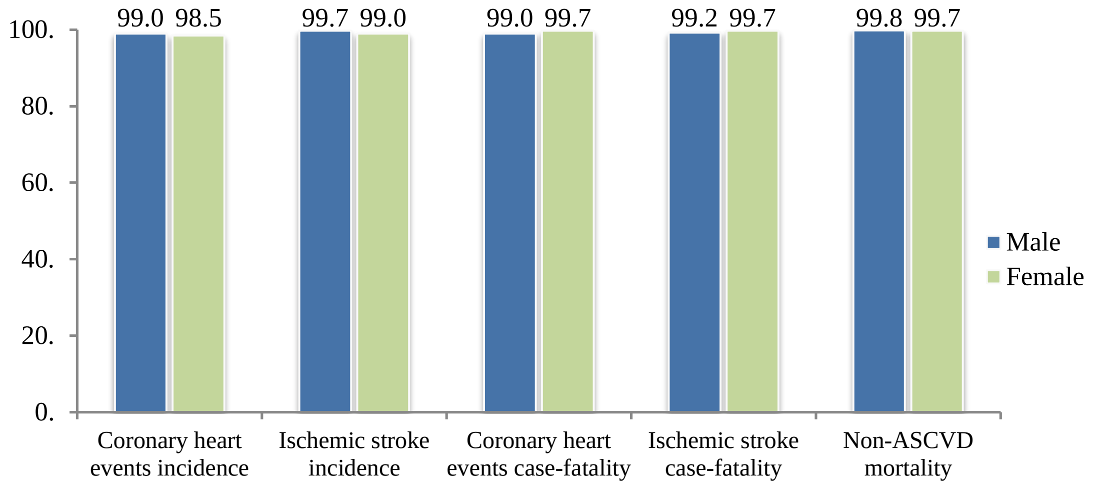

ASCVD, atherosclerotic cardiovascular disease

ONLINE FIGURE 4. Cost-effectiveness of statin treatment among people with different risk of ASCVD. Data are incremental cost-effectiveness ratios (Chinese Yuan (RMB) per QALY gained)

ASCVD Risk	Low risk	Moderate risk	High risk	Low risk	Moderate risk	High risk
**High price scenario before the new policy**						
**Health-care sector perspective**	3,204,000	835,000	499,000	3,935,000	968,000	586,000
**Societal perspective**	3,086,000	756,000	418,000	3,804,000	886,000	504,000
**Lower price scenario (price from new centralized medicine procurement policy)**						
**Health-care sector perspective**	1,347,000	327,000	182,000	1,230,000	276,000	153,000
**Societal perspective**	1,229,000	248,000	101,000	1,099,000	195,000	70,000

Cost-effective	ICER was in the range from ￥64,645 to ￥193,932 RMB
Not cost-effective	ICER ≥￥193,933 RMB

ONLINE FIGURE 5. Prevented ASCVD number and incidence of statin induced-adverse events after implementing the statin intervention strategy for 10 years

Figure S5a Low-dose statin treatment

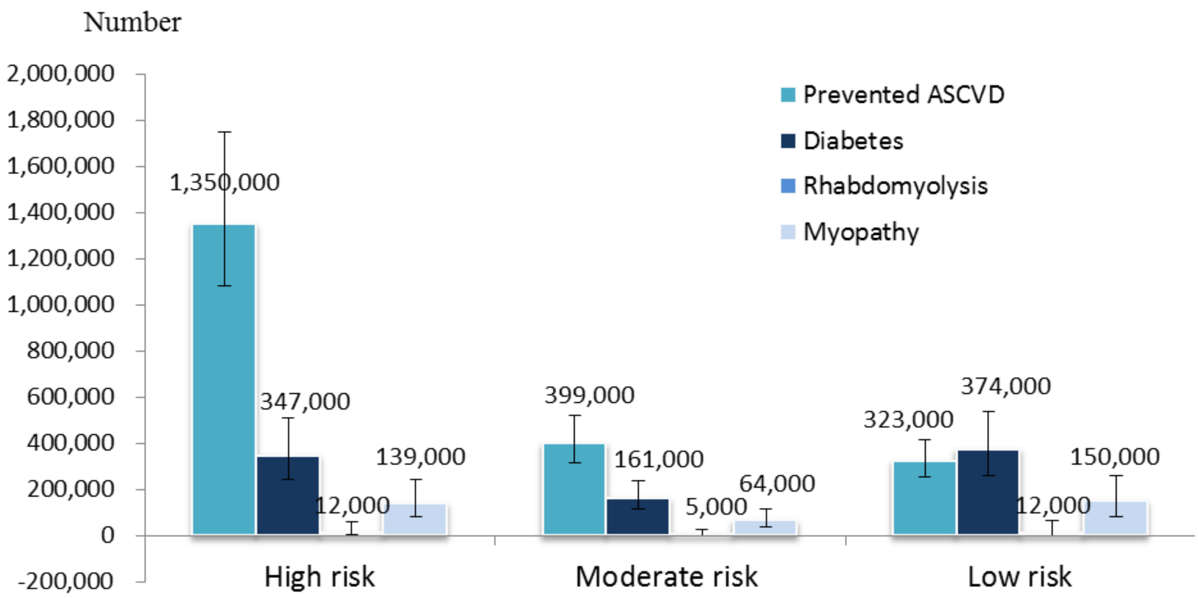

35-64 yeas old	High risk	Moderate risk	Low risk
National population number (N)	80,877,000	88,965,000	407,850,000
Number eligible for intervention (N)	59,525,000	27,579,000	62,809,000

Figure S5b Moderate-dose statin treatment

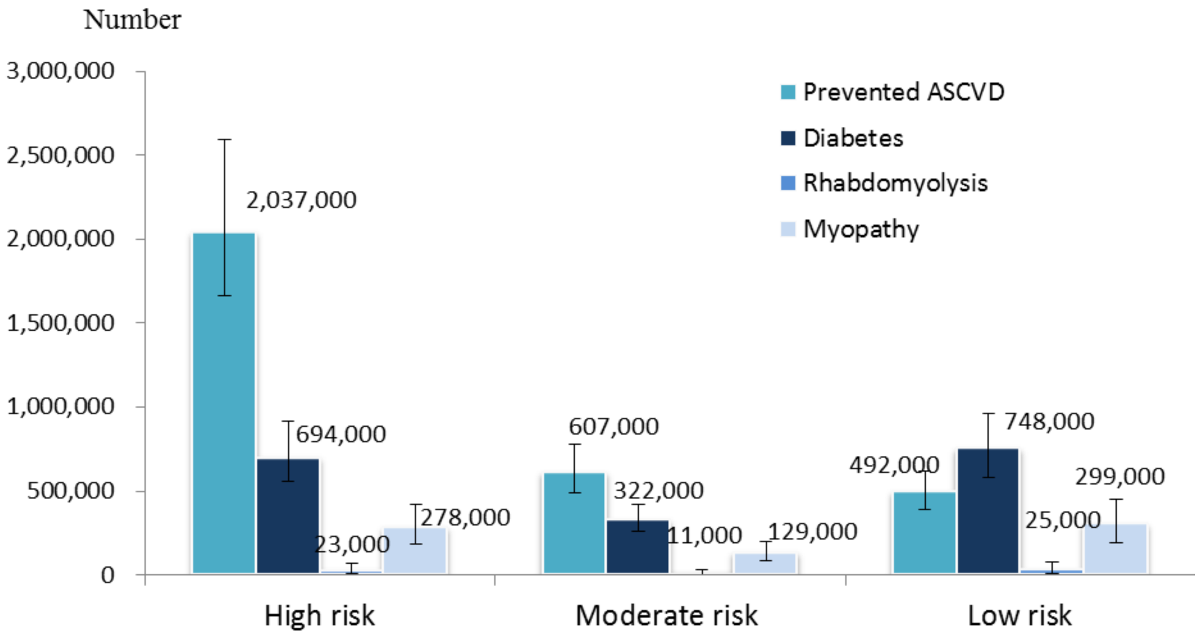

35-64 yeas old	High risk	Moderate risk	Low risk
**National population number (N)**	80,877,000	88,965,000	407,850,000
**Number eligible for intervention (N)**	59,525,000	27,579,000	62,809,000

ASCVD, atherosclerotic cardiovascular disease
I bar represents for the 95% interval confidence.

Online Tables

ONLINE TABLE 1. Baseline characteristics of participants in Chinese Multi-provincial Cohort Study

Variable	High risk	Moderate risk	Low risk	Total
Number (N,%)	2980 (14.0%)	3280 (15.4%)	15005 (70.6%)	21265 (100%)
Female (%)	29.3	38.4	54.2	48.3
10-year risk (%)	10.7 ± 7.1	6.9 ± 1.3	1.8 ± 1.3	3.8 ± 4.4
Age, year	54 ± 7	55.4 ± 5.5	44.4 ± 6.7	47.4 ± 8.1
BMI, Kg/m2	25.1 ± 3.4	25.0 ± 3.4	23.7 ± 3.1	24.1 ± 3.3
SBP, mm Hg	141.7 ± 25.6	139.7 ± 18.8	116.3 ± 14.4	123.5 ± 20.4
DBP, mm Hg	88.2 ± 14	87.4 ± 11.7	77.2 ± 10.2	80.3 ± 12
TC, mg/dL	209.7 ± 51.1	192.0 ± 34	177.2 ± 32.4	184.1 ± 37.7
LDL-C, mg/dL	131.3 ± 48	114.6 ± 32.3	99.3 ± 31.4	106.0 ± 36.1
HDL-C, mg/dL	49.9 ± 14.2	50.6 ± 13.0	55.4 ± 14.3	53.9 ± 14.3
Glucose,mg/dL	118.6 ± 46.2	91.4 ± 15.3	89.4 ± 16.4	93.8 ± 25.0
Diabetes, %	44.9	0.4	1.3	7.3
Hypertension, %	61.7	59.5	17.7	30.3
High TC, %	24.7	9.5	3.7	7.5
High LDL-C,%	23.1	8.6	3.4	6.9
Low HDL-C, %	23.6	20.1	11.9	14.8
Obesity,%	17.6	16.9	9.0	11.5
Current smoking, %	43.5	35.1	25.0	29.1
Lipid-lowering drugs,%	3.1	2.3	0.8	1.3
Eligible for intervention (%)	73.6	31.0	15.4	26.0

BMI, body mass index; DBP, diastolic blood pressure; HDL-C, high-density lipoprotein; LDL-C, low density lipoprotein cholesterol; TC, total cholesterol; SBP, systolic blood pressure.

ONLINE TABLE 2. Indirect costs for model input

Category	Mean	Variance (SE)	Lower limited of 95% CI	Upper limited of 95% CI	Distribution
Patient-time costs due to each hospitalization for ACE (Int$/visit)	521.8	21.1	480.4	563.2	Gamma
Patient-time costs due to each hospitalization for ischemic stroke (Int$/visit)	649.2	26.2	597.8	700.6	Gamma
Yearly patient-time costs due to outpatient clinic visit for ACE or ischemic stroke (Int$/year)	364.0	14.7	335.2	392.8	Gamma
Unpaid caregiver-time costs due to each hospitalization for ACE (Int$/visit)	521.8	21.1	480.4	563.2	Gamma
Unpaid caregiver-time costs due to each hospitalization for ischemic stroke (Int$/visit)	649.2	26.2	597.8	700.6	Gamma
Yearly unpaid caregiver-time costs due to outpatients clinic visit for ACE (Int$/year)	24.0	1.0	22.0	26.0	Gamma
Yearly unpaid caregiver-time costs due to outpatients clinic visit for ischemic stroke (Int$/year)	131.8	5.3	121.4	142.2	Gamma
Transportation costs for each single trip to outpatients clinic (Int$/visit)	2.3	0.1	2.1	2.5	Gamma
Yearly labor market earnings lost due to disability for each ACE patient (Int$/year)	1045.2	42.2	962.5	1127.9	Gamma
Yearly labor market earnings lost due to disability for each ischemic stroke patient (Int$/year)	5732.7	231.6	5278.8	6186.6	Gamma
Yearly labor market earnings lost due to premature death due to ACE or ischemic stroke (Int$/year)	15836.2	639.8	14582.2	17090.2	Gamma

ACE, acute coronary events; CI, confidence interval; SE, standard error. 1 Int$= 3.539. Chinese Yuan (RMB) and all costs were inflated to 2019.

ONLINE TABLE 3. Equations for estimating indirect costs in the model

Category	Loss party	Formulas for indirect cost estimation
**Patient-time costs**		
Hospitalization (Int$/time)	Patient	Daily wages(Int$ /day)×duration of hospitalization(day/admission)×(1-Unemployment rate)
Outpatient Clinic visit (Int$/year)	Patient	Daily wages (Int$ /day)×0.5(day/visit)× 12(visit/year)×Survival year after onset of ASCVD× (1-Unemployment rate)*
**Unpaid caregiver-time costs**		
Hospitalization (Int$/time)	Family number	Daily wages(Int$ /day)×Duration of hospitalization(day/visit)×(1-Unemployment rate)
Outpatient Clinic visit (Int$/year)	Family number	Daily wages(Int$ /day)×0.5(day/visit)× 12(visit/year)×Survival year after onset of ASCVD×Disability rate×(1-Unemployment rate)
**Transportation costs**		
Yearly outpatient clinic visit (Int$/year)	Patient	Single trip cost(Int$ /trip)×2(trip/visit)×12(visit/year)×survival year after onset of ASCVD(year)
**Labor market earnings lost for each patient**		
Disability (Int$/year)	Patient	Average yearly salaries(Int$ /year)×survival year after onset of ASCVD×disability rate(all patients)× (1-unemployment rate)
Premature death (Int$/year)	Dead patient	Average yearly salaries(Int$ /year)×life year loss due to death (year)×(1-unemployment rate)

*, It was estimated that each visit would spend half day and the 12 visits was determined based on the criterion that the prescription interval for chronic disease in China (one month).

ONLINE TABLE 4. Model input parameters of utility and time

Category	Mean	Source
**Utility(QALY weight)**		
Non-fatal ASCVD in the acute stage	0.5	Systematic review of health-related quality of life for disease in China and Global Burden of Disease Study 2016
Non-fatal coronary heart disease in the chronic stage †	0.87	
Non-fatal ischemic stroke in the chronic stage †	0.9	
Diabetes‡	0.951	
Rhabdomyolysis‡	0.883	
Myopathy‡	0.977	
**Time (years)**		
Average time from health to onset of first ACE	5.3	Chinese Multi-provincial Cohort Study
Average time from health to onset of first ischemic stroke	5.4	
Average time from health to non-ASCVD death	5.4	
Average time of chronic stage of ACE	2.9	
Average time of chronic stage of ischemic stroke	3.7	

ACE, acute coronary events, ASCVD, atherosclerotic cardiovascular disease; QALY, quality-adjusted life-year.
*, The adverse events rates were observed from moderate-dose statin treatment, the rates would decline by half for low-dose statin treatment.
†, Data were from systematic review of health-related quality of life for disease in China.
‡, Data were from Global Burden of Disease Study 2016.

ONLINE TABLE 5. Effectiveness and cost-effectiveness of 10-year statin treatment among Chinese adults stratified by ASCVD risk in Chinese Multi-provincial Cohort Study population

	Low-dose statin strategy*			Moderate-dose statin strategy†		
**ASCVD Risk**	**Low**	**Moderate**	**High**	**Low**	**Moderate**	**High**
Relative change of ASCVD incidence among each risk category (%)	-4.1 (-5.2, -3.2)	-6.4 (-8.3, -5.1)	-15.5 (-20.2, -12.2,)	-6.2 (-7.8, -4.9)	-9.7 (-12.3, -7.8)	-23.4 (-29.8, -19.1)
Relative change of ASCVD incidence among whole population (%)	-1.4 (-1.8, -1.1)	-1.7 (-2.3, -1.4)	-5.9 (-7.7, -4.7)	-2.1 (-2.7, -1.7)	-2.7 (-3.4, -2.2)	-8.9 (-11.3, -7.3)
Gained QALY	8.8 (6.4, 11.8)	13.4 (10.6, 17.6)	45.5 (36.4, 59.2)	12.1 (8.7, 16.2)	19.8 (15.7, 25.4)	67.1 (54.5, 85.5)
NNT10	193.0 (150.6, 246.0)	67.3 (51.7, 84.4)	42.8 (33.0, 53.4)	126.8 (101.0, 159.1)	44.3 (34.7, 54.6)	28.4 (22.3, 34.8)
**Statins prices from the new centralized medicine procurement policy (Low statins price scenario)**						
**Societal perspective (Both direct and indirect costs were included)**						
ICER (Int$/QALY)	347,400	70,000	28,500	310,600	55,100	19,800
ICER (RMB/QALY)	1,229,000	248,000	101,000	1,099,000	195,000	70,000
Probability cost saving	0	0	0	0	0	0
Probability highly cost-effective	0	0	11.7	0	0	43.4

Probability cost-effective	0	16.2	87.6	0	50.7	56.6
Probability harmful	0	0	0	0	0	0
Health-care sector perspective (Only direct costs were included)						
ICER (Int$/QALY)	380,700	92,300	51,300	347,500	78,000	43,100
ICER (RMB/QALY)	1,347,000	327,000	182,000	1,230,000	276,000	153,000
Probability cost saving	0	0	0	0	0	0
Probability highly cost-effective	0	0	0	0	0	0
Probability cost-effective	0	0.2	64.9	0	2.6	90.8
Probability harmful	0	0	0	0	0	0
**Higher statins price observed before the new medicine procurement policy were used (High statins price scenario)**						
Societal perspective (Both direct and indirect costs were included)						
ICER (Int$/QALY)	872,000	213,700	118,200	1,075,000	250,400	142,300
ICER (RMB/QALY)	3,086,000	756,000	418,000	3,804,000	886,000	504,000
Probability cost saving	0	0	0	0	0	0
Probability highly cost-effective	0	0	0	0	0	0.1
Probability cost-effective	0	0.1	7.2	0	0.2	4.9
Probability harmful	0	0	0	0	0	0
Health-care sector perspective (Only direct costs were included)						
ICER (Int$/QALY)	905,400	236,000	141,000	1,111,900	273,400	165,600

ICER (RMB/QALY)	3,204,000	835,000	499,000	3,935,000	968,000	586,000
Probability cost saving	0	0	0	0	0	0
Probability highly cost-effective	0	0	0	0	0	0
Probability cost-effective	0	0	0.8	0	0	0.5
Probability harmful	0	0	0	0	0	0

ASCVD, atherosclerotic cardiovascular disease; NNT10, number needed to treat for 10-year intervention; QALY, quality adjusted life year

1 Int$ = 3. 539Chinese Yuan (RMB)

*, Low-dose statin = Simvastatin 20 mg/day, atorvastatin 10mg/day or rosuvastatin 5mg/day

†, High-dose statins = Simvastatin 40 mg/day, atorvastatin 20mg/day or rosuvastatin 10mg/day

High risk: 1) LDL-C ≥ 4.9 moml/L or total cholesterol (TC) ≥ 7.2 mmol/L; 2) diabetes and LDL-C in the range of 1.8-4.8 mmol/L (or TC 3.1-7.2 mmol/L) and age≥40 years; 3) 10-year ASCVD risk ≥10%.
Moderate risk: 10-year ASCVD risk among 5-9.9%.
Low risk: 10-year ASCVD risk ≤ 4.9%.
Cost saving: the saving costs due to decrease of ASCVD was higher than the costs input for statin treatment.
Highly cost-effective: cost per QALY gained was in the range between 0 and Int$ 18,266
Cost-effective: cost per QALY gained was in the range between Int$ 18,267 and Int$ 54,798
Harmful: the lost QALYs due to statin-induced adverse events were larger than the gained QALYs due to decrease of ASCVD

ONLINE TABLE 6. Mean 10-year incremental health-care cost (Int $) of statin vs. no statin treatment for each eligible person stratified by ASCVD risk

	Low-dose statin strategy*			Moderate-dose statin strategy†		
**ASCVD Risk**	**Low risk**	**Moderate risk**	**High risk**	**Low risk**	**Moderate risk**	**High risk**
**Health-care sector perspective**						
Statin prices from the new centralized medicine procurement policy	1,500 (1,200, 1,800)	1,200 (1,000, 1,600)	1,100 (900, 1,400)	1,800 (1,400, 2,300)	1,600 (1,200, 2,000)	1,400 (1,100, 1,800)
High-price staitns before the new policy	3,400 (1,800, 5,900)	3,200 (1,600, 5,600)	3,000 (1,500, 5,300)	5,900 (2,700, 10,100)	5,500 (2,500, 9,800)	5,200 (2,300, 9,500)
**Societal perspective**						
Statin prices from the new centralized medicine procurement policy	1,300 (1,000, 1,700)	900 700, 1,300)	600 (300, 900)	1,600 (1,200, 2,100)	1,100 (700, 1,500)	600, (200, 1,000)
High-price staitns before the new policy	3,200 (1,600, 5,700)	2,600 (1,300, 5,200)	2,500 (1,000, 4,800)	5,700 (2,500, 9,900)	5,000 (2,000, 9,800)	4,500 (1,600, 8,700)

*, simvastatin 20 mg/day, atorvastatin 10mg/day or rosuvastatin 5mg/day
†, simvastatin 40 mg/day, atorvastatin 20mg/day or rosuvastatin 10mg/day
Costs are in 2019 International dollars (Int$). 1 Int$ = 3. 539 Chinese Yuan (RMB)

ONLINE TABLE 7. Results of one-way sensitivity analysis base on the statin price from the new centralized medicine procurement policy and societal perspective

ICER (Int$/QALY)	Low-dose statin strategy			Moderate-dose statin strategy		
ASCVD Risk	Low	Moderate	High	Low	Moderate	High
**Main simulation**	347,400	70,000	28,500	310,600	55,100	19,800
**Available lowest price of statins from the new policy***	330,500	65,400	25,600	286,000	48,800	15,800
**Costs of adverse events treatment**						
Cost of rhabdomyolysis treatment increased by 25%	347,400	70,000	28,500	310,600	55,100	19,800
Cost of rhabdomyolysis treatment decreased by 25%	347,300	70,000	28,500	310,600	55,000	19,800
Cost of diabetes treatment increased by 25%	348,200	70,200	28,700	311,900	55,400	20,000
Cost of diabetes treatment decreased by 25%	346,500	69,700	28,400	309,300	54,700	19,600
**Incidence of adverse events changed**						
Lower limit of rate of rhabdomyolysis (0%)	347,200	69,900	28,500	310,300	55,000	19,700
Upper limit of rate of rhabdomyolysis (0.1%)	348,100	70,100	28,600	311,200	55,200	19,800
Rate of rhabdomyolysis increased by 10 times (0.2)%	349,000	70,300	28,700	313,000	55,500	20,000
Lower limit of rate of myopathy (0.13%)	347,300	70,000	28,500	310,500	55,100	19,800
Upper limit of rate of myopathy (0.41%)	347,500	70,000	28,500	310,700	55,100	19,800
Rate of myopathy increased by 10 times (2.2)%	348,800	70,100	28,500	312,500	55,100	19,800
Lower limit of rate of	318,600	68,000	27,900	281,700	53,400	19,300

diabetes (0.42%)						
Upper limit of rate of diabetes (0.85%)	396,600	72,800	29,400	351,400	57,100	20,400
Rate of diabetes increased by 10 times (6.0)%	Harmful	287,800	63,400	Harmful	3,838,600	73,100
**QALY weight changed†**						
QALY weight of diabetes increased from 0.951 to 0.99993	269,500	64,800	27,200	218,700	49,600	18,500
QALY weight of diabetes decreased from 0.951 to 0.8	3,196,000	93,100	33,900	Harmful	83,000	25,100
QALY weight of diabetes increased from 0.951 to 1	269,400	64,800	27,200	218,600	49,600	18,500

ASCVD, atherosclerotic cardiovascular disease; ICER, incremental cost effectiveness ratios; QALY, quality adjusted life year
*, Costs were estimated from societal perspective and using lowest statin prices from the new centralized medicine procurement policy.
†, QALY weights were from former published paper on statin cost-effectiveness.

ONLINE TABLE 8. Reimbursement policy for outpatient service in the urban and rural resident medical insurance scheme in selected cities of China

No.	Province/ Municipality	City	Reimbursement cap line per year of the coordination policy* (Int$)	Special reimbursement policy for certain diseases	Reimbursemt ratio of the coordination policy			Release year of the files	Resource
					Primary care facilities	Secondary hospitals	Tertiary hospitals		
1	Beijing	Beijing	848	Yes	55%	50%	50%	2017	http://rsj.beijing.gov.cn/xxgk/zcjd/201912/t20191206_946215.html
2	ShangHai	Shanghai	Not mentioned	Not mentioned	70%	60%	50%	2015	https://baijiahao.baidu.com/s?id=1651170119053768088&wfr=spider&for=pc
3	Chongqing	Wushan	8 - 14	Yes	60%	60%	60%	2016	http://wush.cq.gov.cn/info/1212/16511.htm;http://www.cqws120.cn/jydh/ybcs/4620.html
4	Tianjin	Tianjin	Not mentioned	Yes	55-65%	50-60%	45-55%	2014	http://hrss.tj.gov.cn/ecdomain/framework/tj/gnnknhmjegifbbodkjajlpafcampibii/innplgdkegifbbodkjajlpafcampibii.do?isfloat=1&fileid=20140114090902953&moduleIDPage=innplgdkegifbbodkjajlpafcampibii&siteIDPage=tj&pageID=gnnknhmjegifbbodkjajlpafcampibii
5	Zhejiang	Hangzhou	Not mentioned	Yes	60%	50%	30%	2017	http://www.hangzhou.gov.cn/art/2018/1/4/art_1256295_14794809.html

6	Zhejiang	Ningbo	Not mentioned	Yes	50-60%	35-45%	20-30%	2015	http://nbzh.zjzwfw.gov.cn/art/2018/8/3/art_1509771_218.html
7	Guangdong	Guangzhou	14 per month	Yes	70%	50%	50%	2017	http://rsj.gz.gov.cn/zwgk/zwwgk/jcgk/content/post_2409884.html；http://www.gz.gov.cn/gzswjk/2.2.59/201511/39ebdbaa0114407eb2dac5f10c4962aa.shtml?from=singlemessage
8	Guangdong	Shenzhen	283	Yes	60-80%	--	--	2014	http://www.gd.gov.cn/zwgk/zcfgk/content/post_2530804.html
9	Jiangsu	Nanjing	85 - 565	Yes	50%	30%	30%	2018	http://www.njhrss.gov.cn/njsrlzyhshbzj/201810/t20181026_1215840.html
10	Sichuan	Chengdu	57	Yes	60%	60%	60%	2019	http://jcpt.chengdu.gov.cn/dayixian/denglongshequ/detail.html?url=/dayixian/denglongshequ/3001030105/478051_cundetail.html
11	Sichuan	Deyang	28 -57	Yes	75%	75%	75%	2017	http://www.deyang12345.gov.cn/ResultQuery/Detail?menuid=menu_hotspot&id=ODE1MDE@;http://www.deyang.gov.cn/gk/cdzd/ggqsy/yl/913730.htm
12	Yunnan	Kunming	113	Yes	50%	--	--	2012	http://www.km.gov.cn/c/2013-07-17/593475.shtml
13	Anhui	Hefei	28 -565	Yes	60%	---	--	2020	http://www.hefei.gov.cn/xxgk/zcwj/szfwj/104637359.html;http://ah.people.com.cn/n2/2019/0611/c358428-33025943.html

14	Anhui	Wuhu	21	Yes	50%	50%	--	2019	http://credit.wuhu.gov.cn/news/1562056061687.html
15	Fujian	Xiamen	Not mentioned	No information	60%	50%	40%	2014	http://ylbz.xm.gov.cn/zwgk/zfxxgk/ml/zcwj/zcfg/201707/t20170718_1741622.htm
16	Hebei	Shijiazhuang	283	Yes	60%	60%	60%	2019	http://www.gc.gov.cn/col/1531384690314/2019/11/01/1572576801500.html;https://www.sohu.com/a/313996921_376014
17	Hebei	Langfang	Not mentioned	Yes	60%	60%	60%	2016	http://xgc.ncist.edu.cn/article/2018-4-20/art22932.html
18	Heilongjiang	Haerbin	283 -339	Yes	70-75%	65-70%	50-55%	2018	http://hlj.people.com.cn/n2/2018/0102/c220075-31094837.html
19	Heilongjiang	Mudanjiang	28 - 283	Yes	90%	70%	70%	2019	http://xy.mdj.gov.cn/zcfg/bszcfg/201912/t20191221_60075.html；http://zwgk.mdj.gov.cn/bmxxgk/rsj/201812/t20181212_266359.html
20	Henan	Zhengzhou	Not mentioned	Yes (339 int$)#	65%	55%	45%	2016	http://public.zhengzhou.gov.cn/15BAD/475209.jhtml http://public.zhengzhou.gov.cn/02XB/2036672.jhtml
21	Henan	Luoyang	73	Yes	50%	50%	50%	2020	http://haly.si.gov.cn/synrgml/zhengcefagui/zhengcewenjian/yiliaobaoxian2/20200109141512918130.html;http://haly.si.gov.cn/synrgml/zhengcefagui/zhengcewenjian/yiliaobaoxian2/201612291430720058.html
22	hubei	Wuhan	113	Yes	50%	50%	50%	2017	http://www.wh.gov.cn/hbgovinfo/zwgk/szfxxgkml/fggw/gfxwj/201804/t20180404_195215.html

23	Hubei	Huangshi	85 -113	Yes	60%	60%	60%	2017	http://www.huangshi.gov.cn/xxxgk/wjzl/201709/t20170907_314976.html http://www.huangshi.gov.cn/xbmfw/sbfw/yilbx/201411/t20141120_331039.html
24	Hunan	Changsha	226	Yes	60%	Not mentioned	Not mentioned	2018	http://www.csx.gov.cn/csx/zwgk/xxgkml/bmxxgkml/xrsj/tzgg72/2302908/index.html
25	Hunan	Hengyang	170	Yes	50%	50%	50%	2018	http://hunan.sina.com.cn/hy/news/2018-10-18/detail-ihmrasqr9244109.shtml http://cs.bendibao.com/live/20171211/51044.shtm
26	Jilin	Changchun	283	Yes	50%	50%	50%	2019	http://ccyb.changchun.gov.cn/zcfg/zcjd/201912/t20191202_2050501.html
27	Jiangxi	Nanchang	17	Yes	60%	60%	60%	2019	http://www.xinjian.gov.cn/Item/104011.aspx
28	Jiangxi	fuzhou	226	Yes	68%	40%	Not mentioned	2018	http://www.jxlc.gov.cn/art/2018/6/4/art_1559_1080551.html
29	Liaoning	shenyang	23 per month	Yes	55%	55%	55%	2018	http://ybzx.shenyang.gov.cn/newsopen.asp?id=100340
30	Liaoning	Dalian	57	Yes	50%	50%	50%	2019	http://dl.sina.com.cn/news/m/2019-11-19/detail-iihnzahi1835082.shtml http://www.swj.dl.gov.cn/html/2011-10/11734.html

31	Shandong	Jinan	85	Yes	50%	50%	50%	2014	http://www.zhangqiu.gov.cn/info/t697494695/front/zhengwufuwudating/sanji/serverhallthree1.aspx?id=30 http://jn.bendibao.com/live/20191225/52331.shtm
32	Shandong	Qingdao	85 - 203	Yes	75-80%	65-70%	55-65%	2019	http://ybj.qingdao.gov.cn/n28356081/n32567782/n32567784/n32567792/190912165033646578.html
33	Shaanxi	Xian	141	Yes	70-80%	50%	50%	2007, 2016	http://www.xa.gov.cn/web_files/attachment/201702/24/2017_02_24_308_NLIEUT_22688.PDF http://www.xatao029.com/yanta/2291.html
34	Shanxi	Lvliang	Not mentioned	Yes	No details	No details	No details	2017	http://www.lvliang.gov.cn/llxxgk/zfxxgk/xxgkml/szfwj/201707/t20170725_291503.html
35	Shanxi	Taiyuan	57	Yes	80%	80%	80%	2017	http://shanxi.sina.com.cn/zimeiti/2017-08-10/detail-ifyixhyw6584529.shtml http://www.taiyuan.gov.cn/doc/2017/09/30/175916.shtml
36	Gansu	Lanzhou	28	Yes	70%	70%	750%	2019	http://credit.lanzhou.gov.cn/323.news.detail.dhtml?news_id=44054 http://www.lzush.com.cn/item/25388.aspx
37	Gansu	Tianshui	Not mentioned	Yes	Not mentioned	Not mentioned	Not mentioned	2019	http://zwgk.zjc.gov.cn/index.php?m=content&c=index&a=show&catid=207&id=13666 http://www.ts407yy.cn/html/2019/xlhbx_1225/644.html
38	Hainan	Haikou	Not mentioned	Yes	90%	75%	65%	2019	http://www.haikou.gov.cn/zxfw/smfw/sb/sbzx/201911/t20191125_1466736.html

39	Hainan	Sanya	85	Yes	60%	50%	Not Mentioned	2019	http://www.sanya.gov.cn/sanyasite/sbcjwt/202001/edb0a9df348c4c2fafc912062a0d0e05.shtml
40	Guizhou	Zunyi	17	Yes	50%	50%	50%	2017	http://www.zunyi.gov.cn/bsfw/cjwt/201710/t20171024_10297568.html http://zy.gzegn.gov.cn/art/2018/8/31/art_42653_520923.html
41	Qinghai	Xining	34	Yes	50%	Not Mentioned	Not Mentioned	2019	https://baijiahao.baidu.com/s?id=1652698696971469277&wfr=spider&for=pc
42	Guangxi	Nanning	57	Yes	75%	Not Mentioned	Not Mentioned	2017	http://ybj.nanning.gov.cn/xxgk_164/wjzl/zzqwj/t2838517.html
43	Neimenggu	Hohhot	565	Yes	65%	60%	50%	2019	http://www.huhhot.gov.cn/ylbzj/xxgk/bmxxgkml/201909/t20190929_563070.html http://law.51labour.com/lawshow-100551.html
44	Ningxia	Yinchuan	73	Yes	50-65%	Not Mentioned	Not Mentioned	2019	http://www.yinchuan.gov.cn/xxgk/bmxxgkml/ycsylbzj/xxgkml_38315/zcjd_39128/201911/t20191112_1846939.html http://www.cpic.com.cn/c/2017-11-02/1229308.shtml http://www.yinchuan.gov.cn/xwzx/mrdt/201711/t20171103_550933.html
45	xizang	Lhasa	Not Mentioned	Yes	60%	60%	60%	2019	www.lasa.gov.cn/lasa/xwzx/201912/9ecc605828ec4626a4ad78d89ef892e5.shtml http://www.xzfkyy.cn/ylfw/jyzn/ybfw/zzqybjsb/

46	Xinjiang	Urumchi	141	Yes	50%	50%	50%	2015	http://www.xinjiangnet.com.cn/2015/1026/ 1478982.shtml

*, Reimbursement cap line was the maximum amount cost paid by the medical insurance funds.
#, The city had special reimbursement policy character as high both reimbursement rate and reimbursement cap line for outpatient treatment fee of certain diseases, and the special policy covered dyslipidemia, other did not. The cities were selected non-randomisedly.

ONLINE TABLE 9. Risk factor level in recent national survey and baseline survey of CMCS participants

Risk factors	Age group	National survey	CMCS
SBP (mmHg)	45-59 years	134	127
DBP (mmHg)	45-59 years	79	80
TC (mg/dl)	45-59 years	185	183
HDL-C (mg/dl)	45-59 years	46	54
Fasting blood glucose (mg/dl)	40-49 years	101	93
	50-59 years	106	96
Smoking (%)	--	28.1 (≥15 years)	29.1 (35-64 years)

CMCS, Chinese Multi-provincial Cohort Study; DBP, diastolic blood pressure; SBP, systolic blood pressure; TC, total cholesterol; HDL-C, High-density lipoprotein cholesterol

References:

1. X Zhang, J Liu, M Wang, Y Qi, J Sun, J Liu, Y Wang, Y Hao, Y Li, M Zhou, D Zhao. Twenty-year epidemiologic study on LDL-C levels in relation to the risks of atherosclerotic event, hemorrhagic stroke, and cancer death among young and middle-aged population in China. *J Clin Lipidol.* 2018;12(5):1179-1189.
2. C Huang, AE Moran, PG Coxson, X Yang, F Liu, J Cao, K Chen, M Wang, J He, L Goldman, D Zhao, PL Kinney, D Gu. Potential Cardiovascular and Total Mortality Benefits of Air Pollution Control in Urban China. *Circulation.* 2017;136(17):1575-1584.
3. J Liu, Y Hong, RS D'Agostino, Z Wu, W Wang, J Sun, PW Wilson, WB Kannel, D Zhao. Predictive value for the Chinese population of the Framingham CHD risk assessment tool compared with the Chinese Multi-Provincial Cohort Study. *JAMA.* 2004;291(21):2591-2599.
4. W Miao, L Jing, Z Dong. New risk assessment tool of atherosclerotic cardiovascular disease for Chinese adults. *Chinese Journal of Cardiology.* 2018(2):87-91.
5. Joint committee for developing Chinese guidelines on prevention and treatment of dyslipidemia in adults. Guideline on the prevention and treatment of dyslipidemia in Chinese adults (2016-Update). *Chinese Journal of Cardiology.* 2016;44(10):833-853.
6. GD Sanders, PJ Neumann, A Basu, DW Brock, D Feeny, M Krahn, KM Kuntz, DO Meltzer, DK Owens, LA Prosser, JA Salomon, MJ Sculpher, TA Trikalinos, LB Russell, JE Siegel, TG Ganiats. Recommendations for Conduct, Methodological Practices, and Reporting of Cost-effectiveness Analyses: Second Panel on Cost-Effectiveness in Health and Medicine. *JAMA.* 2016;316(10):1093-1103.
7. Y Hao, J Liu, J Liu, N Yang, SJ Smith, Y Huo, GC Fonarow, J Ge, KA Taubert, L Morgan, M Zhou, Y Xing, CS Ma, Y Han, D Zhao. Sex Differences in In-Hospital Management and Outcomes of Patients With Acute Coronary Syndrome. *Circulation.* 2019;139(15):1776-1785.
8. Y Yang, YZ Shi, N Zhang, S Wang, GS Ungvari, CH Ng, YL Wang, XQ Zhao, YJ Wang, CX Wang, YT Xiang. The Disability Rate of 5-Year Post-Stroke and Its Correlation Factors: A National Survey in China. *Plos One.* 2016;11(11):e165341.
9. International Labour Organization. Country profiles. The latest decent work statistics by country. https://ilostat.ilo.org/data/country-profiles (accessed 6 Feb 2020).
10. *Ministry of Health of the People's Republic of China.China' Health Statistics Yearbook 2018. Beijing: China Union Medical University Press; 2018.*
11. Integrated Management Platform of Beijing Medicine Sunshine Purchase. http://210.73.89.76/ServiceSelect/GetServiceSelectList. (accessed 5 December 2018).
12. Beijing Medicine Sunshine Purchasing. Notice on announcement of the bidding result of centralized medicine procurement in "4+7" cities. https://yp.bjmbc.org.cn/view/index/detail.html?id=ZDAT00000000000001682913.(accessed 9 April 2019).
13. B Mihaylova, J Emberson, L Blackwell, A Keech, J Simes, EH Barnes, M Voysey, A Gray, R Collins, C Baigent. The effects of lowering LDL cholesterol with statin therapy in people at low risk of vascular disease: meta-analysis of individual data from 27 randomised trials. *Lancet (London, England).* 2012;380(9841):581-590.
14. R Chou, T Dana, I Blazina, M Daeges, TL Jeanne. Statins for Prevention of Cardiovascular Disease in Adults: Evidence Report and Systematic Review for the US Preventive Services Task Force. *JAMA.* 2016;316(19):2008-2024.
15. DS Kazi, JM Penko, K Bibbins-Domingo. Statins for Primary Prevention of Cardiovascular Disease: Review of Evidence and Recommendations for Clinical Practice. *The Medical clinics of North America.* 2017;101(4):689-699.
16. HPS2-THRIVE randomized placebo-controlled trial in 25 673 high-risk patients of ER niacin/laropiprant: trial design, pre-specified muscle and liver outcomes, and reasons for stopping study treatment. *Eur Heart J.* 2013;34(17):1279-1291.
17. T Ta. Statin safety evaluation workgroup. Chinese expert consensus for statin safety. *Chinese Journal of Cardiology.* 2014;42(11):890-894.

18. Z Ting, G Hai-jing, G Guo-en, M Ai-xia. Health-Related Quality of Life for Disease Population in China based on EQ-5D: A Systematic Review. *Chin J Evid-based Med.* 2016(02):135-142.

19. MJ Pletcher, L Lazar, K Bibbins-Domingo, A Moran, N Rodondi, P Coxson, J Lightwood, L Williams, L Goldman. Comparing impact and cost-effectiveness of primary prevention strategies for lipid-lowering. *Ann Intern Med.* 2009;150(4):243-254.

20. LD Lazar, MJ Pletcher, PG Coxson, K Bibbins-Domingo, L Goldman. Cost-effectiveness of statin therapy for primary prevention in a low-cost statin era. *Circulation.* 2011;124(2):146-153.

21. Global, regional, and national incidence, prevalence, and years lived with disability for 328 diseases and injuries for 195 countries, 1990-2016: a systematic analysis for the Global Burden of Disease Study 2016. *Lancet (London, England).* 2017;390(10100):1211-1259.

22. K Hajifathalian, P Ueda, Y Lu, M Woodward, A Ahmadvand, CA Aguilar-Salinas, F Azizi, R Cifkova, M Di Cesare, L Eriksen, F Farzadfar, N Ikeda, D Khalili, YH Khang, V Lanska, L Leon-Munoz, D Magliano, KP Msyamboza, K Oh, F Rodriguez-Artalejo, R Rojas-Martinez, JE Shaw, GA Stevens, J Tolstrup, B Zhou, JA Salomon, M Ezzati, G Danaei. A novel risk score to predict cardiovascular disease risk in national populations (Globorisk): a pooled analysis of prospective cohorts and health examination surveys. *The lancet. Diabetes & endocrinology.* 2015;3(5):339-355.

23. ER Atkins, Du X, Y Wu, R Gao, A Patel, CK Chow. Use of cardiovascular prevention treatments after acute coronary syndrome in China and associated factors. *Int J Cardiol.* 2017;241:444-449.

24. W Longde, Y Ling, H Yang, Z Yi, W Yongjun, J Xunming, N Xiaoyuan, Q Qiumin, H Li, X Yuming, L Mei, S Jiayi, L Jing, Z Dong. Fixed-dose combination treatment after stroke for secondary prevention in China: a national community-based study. *Stroke.* 2015;46(5):1295-1300.

25. Chinese Society of Cardiology of Chinese Medical Association. Chinese guidelines for the prevention of cardiovascular diseases(2017). *Chinese Journal of Cardiology.* 2018(1):10-25.

26. Chinese Society of Cardiology of Chinese Medical Association. Expert consensus document on appropriate use of beta-adrenergic receptor blocker in patients with cardiovascular diseases. *CHINESE JOURNAL OF CARDIOLOGY.* 2009;37(3):195-209.

27. Chinese Society of Cardiology of Chinese Medical Association. Chinese expert consensus document on angiotensin converting enzyme inhibitors in cardiovascular diseases. *CHINESE JOURNAL OF CARDIOLOGY.* 2007;35(2):97-106.

28. Global Burden of Disease Collaborative Network.Global Burden of Disease Study 2017 (GBD 2017) Results.Seattle, United States: Institute for Health Metrics and Evaluation (IHME), 2018. http://ghdx.healthdata.org/gbd-results-tool (accessed 6 Feb 2020).
